# Exposing Zn(002) Texture with Sucralose Additive for Stable and Dendrite-Free Aqueous Zinc-Ion Batteries

**DOI:** 10.1007/s40820-025-01954-3

**Published:** 2026-01-09

**Authors:** Feiyu Tao, Yingke Ren, Li’e Mo, Yifan Wang, Yang Huang, Hong Zhang, Chengwu Shi, Zhaoqian Li, Jiaqin Liu, Lei Chen, Linhua Hu, Yucheng Wu

**Affiliations:** 1 School of New Energy Engineering, Hefei Institute of Technology, Anhui 238076 Hefei, People’s Republic of China; 2 College of Science, Hebei University of Science and Technology, Hebei 050018 Shijiazhuang, People’s Republic of China; 3 Key Laboratory of Photovoltaic and Energy Conservation Materials, CAS, Institute of Solid State Physics, Hefei Institutes of Physical Science, Chinese Academy of Sciences, Anhui 230031 Hefei, People’s Republic of China; 4 School of Environmental and Chemical Engineering, Jiangsu University of Science and Technology, 212003 Zhenjiang, People’s Republic of China; 5 Hebei Computational Optical Imaging and Photoelectric Detection Technology Innovation Center, Hebei International Joint Research Center for Computational Optical Imaging and Intelligent Sensing, School of Mathematics and Physics Science and Engineering, Hebei University of Engineering, Hebei 056038 Handan, People’s Republic of China; 6 School of Chemistry, Beijing University of Chemical Technology, 100029 Beijing, People’s Republic of China

**Keywords:** Aqueous zinc-ion batteries, Zinc anode, Oriented growth, (002) texture

## Abstract

Aqueous zinc-ion batteries (AZIBs) are currently confronted with the challenge of achieving long-term cyclic stability under high current densities. This issue is primarily attributed to the excessive growth of dendrites and the occurrence of significant side reactions. Herein, sucralose (SCL), as an electrolyte additive, has been used to promote the exposure of the Zn(002) texture. The introduction of SCL can adjust the Zn^2+^ nucleation and diffusion along different crystal facets, promoting the exposure of the Zn(002) texture. By substituting water molecules in the [Zn(H_2_O)_6_]^2+^, SCL reconfigures the hydrogen bond network in the electrolyte, reconstructing the solvation structure and suppressing the hydrogen evolution reaction. Consequently, the Zn//Zn symmetric battery exhibits long-term cycling stability of over 4900 h at 1 mA cm^−2^–1 mAh cm^−2^. Even at a harsh condition of 30 mA cm^−2^–30 mAh cm^−2^ (DOD = 73.3%), it can stably cycle for 171 h. The CE of the Zn//Cu half battery reaches 99.61% at 0.2 mA cm^−2^ with 0.2 mAh cm^−2^. Employing the optimized electrolyte, after 500 cycles, a high specific capacity of 420 mAh g^−1^ can be retained for the NH_4_V_4_O_10_//Zn full battery at 500 mA g^−1^, corresponding to a capacity retention of 90.7%.

**Supplementary Information:**

The online version contains supplementary material available at 10.1007/s40820-025-01954-3.

## Highlights

- Sucralose (SCL) has been unveiled as an electrolyte additive to promote the exposure of the Zn(002) texture.
- SCL has been verified to disrupt the solvation structure around zinc ions and reduce water activity on Zn anode.
- After adding SCL additives, Zn//Zn battery achieves the cycling lifespan of 171 h at 30 mA cm^−2^–30 mAh cm^−2^ (DOD = 73.3%). Zn//Cu battery achieves a high Coulombic efficiency of 99.61% at 0.2 mA cm^−2^ with 0.2 mAh cm^−2^.

**Supplementary Information:**

The online version contains supplementary material available at 10.1007/s40820-025-01954-3.

## Graphical abstract

**Figure:**
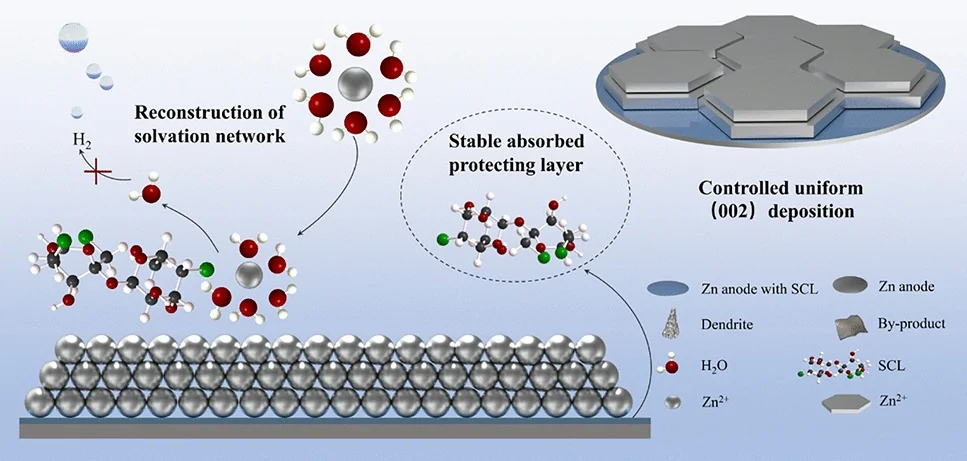

## Introduction

Aqueous zinc-ion batteries (AZIBs) are increasingly recognized as attractive candidates for energy storage applications, owing to their inherent safety, cost-effectiveness, and eco-friendly characteristics [1–4]. Zinc, as an anode material, offers exceptional theoretical specific capacity (820 mAh g^−1^) and volumetric capacity (5855 mAh cm^−3^) [5–7], making it an attractive candidate for grid-scale energy storage applications. Nevertheless, commercial Zn foil cannot work stably under high depth of discharge (DOD) and current density, impeding the widespread commercialization of ZIBs [8]. High DOD leads to an uneven distribution of local Zn^2+^ concentration at the electrode surface, causing 3D Zn deposition with arbitrary orientation, which results in severe dendrites [9–12]. Uneven zinc stripping during the discharge process also exacerbates the formation of holes and further promotes dendrite growth, significantly compromising the integrity and stability of the Zn anode [13]. Under high current densities, except for the generated hydrogen gas, the enhanced hydrogen evolution reaction may affect the local pH environment near the electrode surface, resulting in the generation of detrimental side products that can adversely influence zinc deposition.

Alleviating zinc dendrite growth is primary to improve the stability of ZIBs [14, 15]. Zn, as a hexagonal close-packed metal, exhibits the primary crystal facets of (002), (100), and (101) planes [16]. During deposition, the Zn(101) and (100) facets with larger angles to the substrate often result in uneven zinc deposition. In contrast, if Zn^2+^ is deposited along the Zn(002) crystal plane, the Zn(002) tends to deposit on the substrate in a parallel type, a planar and smooth zinc deposition layer can be achieved [17]. Additionally, the Zn(002) plane, with the lowest surface energy [18], has lower electrochemical activity toward hydrogen evolution reaction (HER) and corrosion reactions [19]. Controlling the preferential crystal orientation is crucial for AZIBs, particularly in suppressing dendrite growth and improving battery stability [20, 21].

Herein, a sweetener, sucralose (SCL), was introduced into the Zn(OTF)_2_ electrolyte to enhance the reversibility of the zinc anode. Due to the strong interaction, the SCL firmly adsorbs on the Zn surface. The as-formed dynamic protection layer can effectively repel active water molecules at the interface, inhibiting water-induced side reactions and expanding the electrochemical window of the electrolyte. After binding with the SCL molecules, the suppressed zinc deposition along the (002) direction leads to a large areal exposure of the (002) facet, which facilitates highly reversible and dendrite-free Zn anodes. Benefiting from the introduction of SCL, the Zn//Zn symmetric battery exhibits prolonged lifespan of 4900 h at 1 mA cm^−2^–1 mAh cm^−2^ and 171 h at 30 mA cm^−2^–30 mAh cm^−2^ (DOD = 73.3%). The CE of the Zn//Cu half battery reaches 99.61% at 0.2 mA cm^−2^ with 0.2 mAh cm^−2^, confirming the high plating/stripping reversibility of the zinc anode. The NH_4_V_4_O_10_//Zn full battery, after being cycled 500 times at 500 mA g^−1^, keeps a specific capacity of 420 mAh g^−1^ (a capacity retention of 90.7%). This work highlights the preferential Zn deposition facilitated by the SCL electrolyte additive for achieving highly reversible AZIBs.

## Experimental Section

### Electrolytes and Cathode Material Preparation

All reagents were used without further purification. The Zn(OTF)_2_ electrolyte was prepared by dissolving 0.1 mol of Zn(CF_3_SO_3_)_2_ (Sigma-Aldrich) in 100 mL of deionized water. The SCL/Zn(OTF)_2_ electrolyte was subsequently formulated by adding 4, 5, and 6 wt% of sucralose to the as-prepared Zn(CF_3_SO_3_)_2_ solution. The cathode material was synthesized via a hydrothermal process. In detail, 7.5 mmol of H_2_C_2_O_4_·2H_2_O (Sinopharm, 99.8%) was dissolved in deionized water at 60 °C. Subsequently, under constant stirring, 10 mmol of NH_4_VO_3_ (Adamasbeta) was incorporated into the solution. The ensuing uniform mixture was then transferred into a 100 mL Teflon-lined autoclave and subjected to heating in a temperature-regulated oven at 180 °C for 24 h. Subsequently, the resulting product was meticulously rinsed with deionized water via centrifugation and then dried at 80 °C.

### Materials Characterization

XRD patterns were collected using a Rigaku Smart Lab 9KW X-ray diffractometer from Japan, operating with Cu-Kα radiation of wavelength 1.540593 nm. FESEM (SU8020) at 10 kV acceleration voltage was used to study the samples’ surface features. XPS analysis was done on a Kratos AXIS Supra + spectrometer, with energies relative to binding calibrated by the C 1* s* peak at 284.8 eV, with the aim of taking into account charging effects on the surface. Surface topography of the zinc electrodes was evaluated using a three-dimensional digital microscope (VK-X250). In situ optical microscopy observations were carried out with a custom-designed open electrochemical cell. The pH values were determined with a Mettler Toledo pH meter. Zeta potential measurements were carried out using a Zeta Sizer NANOPLUS instrument. For zeta potential testing, liquid samples were prepared by ultrasonically dispersing the Zn deposits (obtained at 5 mA cm^−2^ with a capacity of 5 mAh cm^−2^) from Cu foil into 10 mL of electrolyte to form uniform suspensions. All NMR spectra were acquired on a Bruker AVANCE 600 MHz spectrometer (Germany). Raman spectra were obtained using a WITec alpha300R spectrometer equipped with a 532 nm diode-pumped solid-state laser, covering a spectral range from 4000 to 100 cm^−1^.

### Electrochemical Characterization

Potentiostatic current–time transient measurements were conducted under a constant overpotential of -150 mV. LSV was performed on a CHI660E electrochemical workstation. Stainless steel was used as the working electrode, and a zinc plate functioned as both the counter and reference electrodes. The electrolyte’s nucleation overpotential was ascertained through cyclic voltammetry at a scan rate of 1 mV s^−1^. The differential capacitance was determined using the equation: C = − (ω Zₘ)^−1^. A frequency of 1 Hz was used for this calculation. Tafel polarization curves were acquired by sweeping the potential from − 0.5 to 0.5 V at a rate of 5 mV s^−1^ in Zn//Zn symmetric cells. In these symmetric batteries, the zinc foil thickness was 100 μm when tested at 1 mA cm^−2^, while a reduced thickness of 70 μm was used for tests at 30 mA cm^−2^. For Zn//Cu and full batteries, the zinc foil thickness remained at 100 μm. The cathode material used in the full batteries was NH_4_V_4_O_10_. Long-term cycling performance and Coulombic efficiency (CE) were evaluated using a battery test system (BTS 3000, Neware, Shenzhen, China). Activation energy (Eₐ) was derived from electrochemical impedance spectroscopy (EIS) measurements conducted at varying temperatures. This analysis was based on the Arrhenius equation:

1 $${\mathrm{R}}_{\mathrm{ct}}^{-1}=\text{A }{\mathrm{e}}^{-\frac{{\mathrm{E}}_{\mathrm{a}}}{\text{R T}}}$$

### Density Functional Theory Method

Density functional theory (DFT) calculations were performed using the *Vienna *Ab initio Simulation Package (VASP). The exchange–correlation energy was evaluated using the GGA method with the PBE functional. A plane-wave basis set at a 450 eV kinetic energy cutoff was applied in all computations. During structural optimization, ionic forces were relaxed to below 0.02 eV Å^−1^, and the electronic convergence criterion was set at 10^–5^ eV. A five-layer Zn surface supercell containing 64 atoms per layer was used to model the Zn facet. The adsorption energy between the Zn surface and various molecules was calculated using the following expression:

2 $$\Delta {E}_{\mathrm{ad}}={E}_{\mathrm{Zn}-\mathrm{slab}+\mathrm{molecules}}-{E}_{\mathrm{Zn}-\mathrm{slab}}-{E}_{\mathrm{molecules}}$$

The climbing image nudged elastic band (CI-NEB) method, implemented via the VTST Tools within the VASP framework, was employed to determine the migration energy barrier. The migration energy is defined as the relative energy between adjacent high-symmetric zinc adsorption positions along the migration path.

Molecular dynamics simulations were executed via the Forcite module, leveraging the COMPASS II force field for potential energy assessments. These simulations were performed under the NVT ensemble at 298 K, with temperature governed by the Nose–Hoover technique. The energy and force convergence thresholds were established at 1.0 × 10^4^ kcal mol^−1^ and 0.005 kcal mol^−1^ Å^−1^, respectively. A time increment of 1 fs was utilized throughout the simulations.

## Results and Discussion

### Adsorption Behavior and Interfacial Regulation of SCL on Zinc Surface

**Fig. 1:**
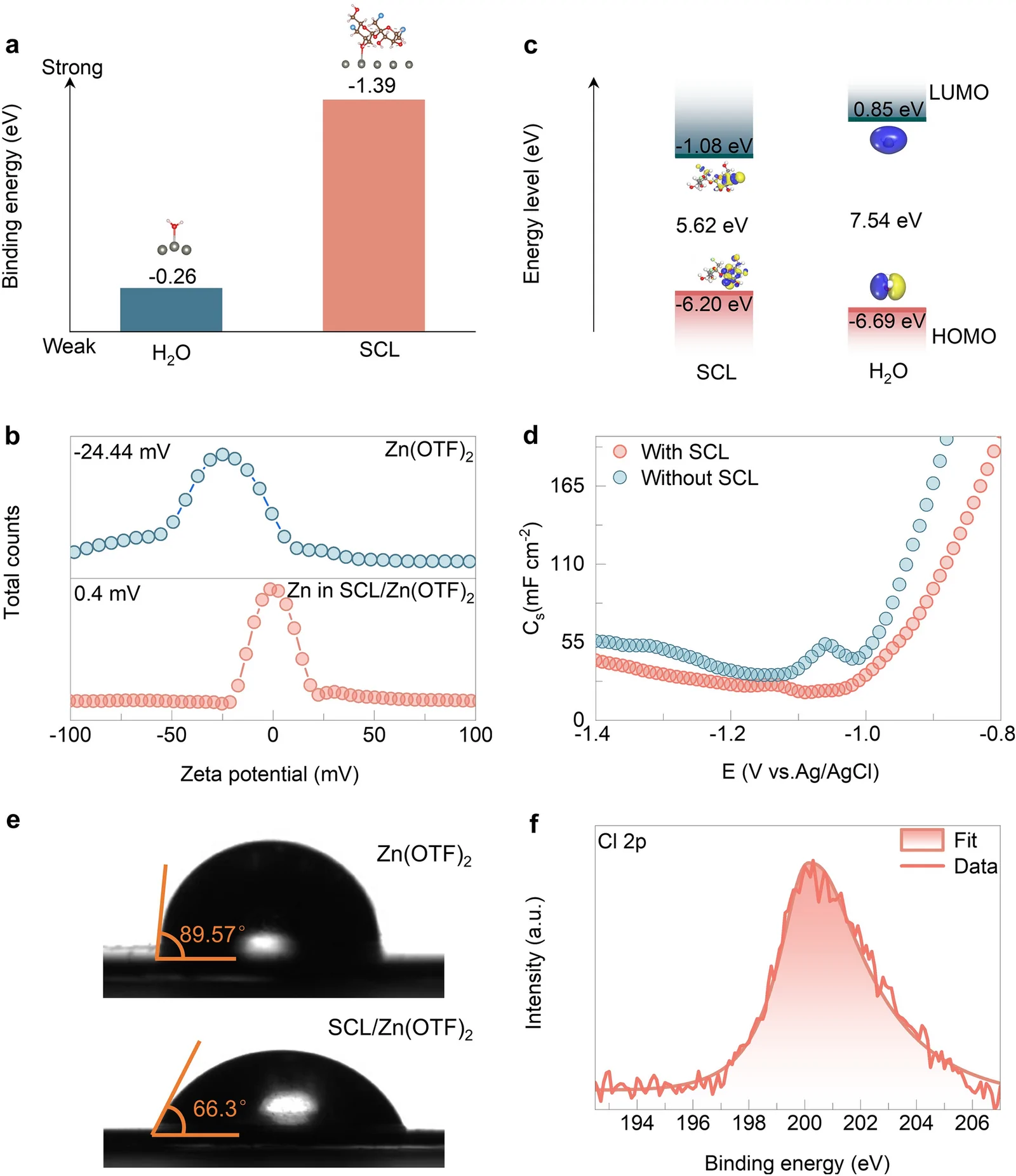
**a** Adsorption energy of H_2_O and SCL molecules on the Zn (002) surface. **b** Zeta potential of zinc powder with and without SCL additive. **c** HOMO and LUMO energy levels of SCL and H_2_O. **d** Differential capacitance curve for Zn in Na_2_SO_4_ solution with/without SCL additive. **e** Contact angles between Zn(OTF)_2_ and Zn foil, SCL/Zn(OTF)_2_ and Zn foil. **f** The Cl 2*p* XPS spectra of Zn in SCL/Zn(OTF)_2_ solution

Figure 1a compares the adsorption energies. Owing to the rich hydroxyl groups (Fig. S1), the SCL/Zn(002) facet shows a lower adsorption energy than the water/Zn(002) facet, suggesting that SCL has a thermodynamic tendency for adsorption onto Zn surfaces. The presence of SCL in the electric double layer (EDL) significantly modifies the charge distribution on the surface of the Zn electrode, leading to reduced aggregation of H_2_O molecules, which can effectively mitigate zinc corrosion and suppress side reactions. In this work, the optimal concentration of SCL is determined to be 5 wt% which is comprehensively discussed and illustrated in Fig. S2. As shown in Fig. 1b, the addition of SCL leads to a substantial increase of zeta potential from -24.44 to 0.4 mV, indicating a significant enhancement in surface charge modulation. Figure S3 shows the 3D differential charge density; the SCL reveals a significant overlap of electron clouds and charge transfer with the Zn surface, indicating the strong interaction between SCL and Zn [22, 23].

Figure 1c shows the energy levels of molecular orbitals. Compared with the H_2_O (− 6.69 eV), the higher HOMO energy level of SCL (-6.2 eV) suggests that SCL is more likely to lose electrons and adsorb onto the zinc surface [24], regulating the interfacial structure, as confirmed by the reduced capacity in Fig. 1d [25]. The electrostatic potential mapping in Fig. S4 further proves that the rich hydroxyl groups of the SCL molecule can offer multiple binding sites for H_2_O and cations [26]. Consequently, the SCL/Zn(OTF)_2_ electrolyte shows better zincophilic features than the Zn(OTF)_2_ electrolyte (Fig. 1e) [27]. After immersing zinc foil in the SCL/Zn(OTF)_2_ electrolyte for a certain time, the XPS data displays characteristic Cl 2*p* peaks at 200 eV corresponding to organo-chlorine species (Fig. 1f) [28], and no peak is observed when using pure Zn(OTF)_2_ (Fig. S5). All the above findings demonstrate that SCL can effectively adsorb onto the zinc surface and regulate the surface structure. As illustrated in Fig. S6, introducing SCL into the Zn(OTF)_2_ electrolyte facilitates the approach of SCL molecules to the inner Helmholtz plane (IHP) of the electric double layer (EDL) at the zinc surface. Owing to the favorable zincophilic characteristics, the SCL molecules will replace some active sites previously occupied by water molecules and the modified Zn/electrolyte interface can suppress side reactions and regulate Zn deposition behavior [29].

### Effect of SCL on Electrolyte Properties

**Fig. 2:**
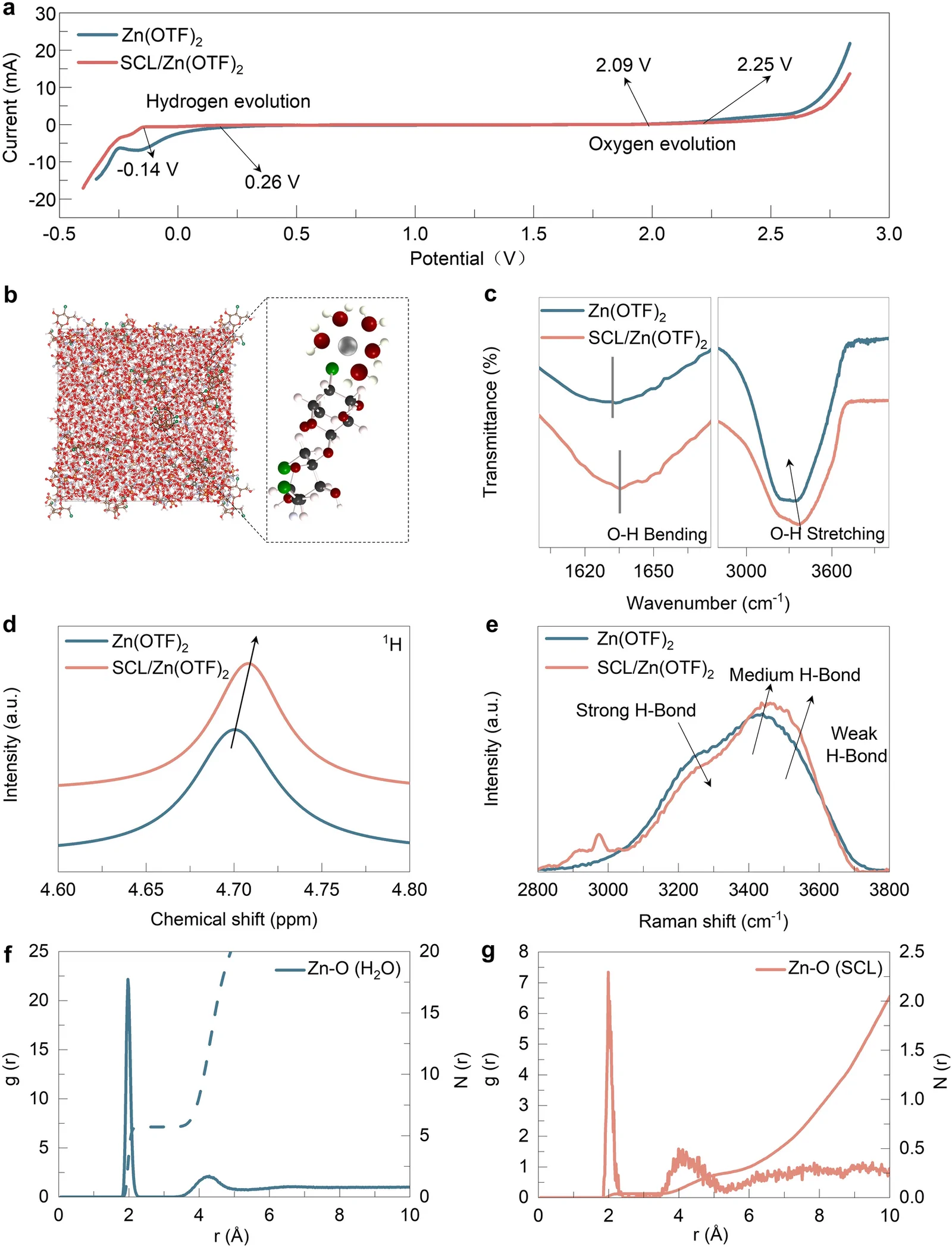
**a** LSV curves in Zn(OTF)_2_ and SCL/Zn(OTF)_2_ electrolytes. **b** Snapshots of the MD simulation of SCL/Zn(OTF)_2_ electrolyte. **c** FTIR spectra of Zn(OTF)_2_ and SCL/Zn(OTF)_2_ electrolytes. **d** ^1^H NMR spectra of different electrolytes. **e** Raman spectrum of different electrolytes. RDFs of **f** Zn–O (H_2_O), **g** Zn–O (SCL) collected from MD simulations in SCL/Zn(OTF)_2_ electrolyte

Figure 2a exhibits the linear sweep voltammetry (LSV) data. Compared with the Zn(OTF)_2_ electrolyte, the SCL/Zn(OTF)_2_ electrolyte shows an onset potential shift from 0.26 to − 0.14 V, indicating the suppression of HER. The increased overpotential for OER further endorses the beneficial impact of the SCL additive in mitigating water decomposition [30]. The Gibbs free energies (ΔG) are employed to quantify the thermodynamic energy barrier associated with the HER occurring on the Zn anode (Fig. S7). The Zn anode in the SCL/Zn(OTF)_2_ electrolyte exhibits a higher energy barrier (ΔG = 1.078 eV) compared to that in the Zn(OTF)_2_ electrolyte (ΔG = 0.992 eV), suggesting that the SCL/Zn(OTF)_2_ electrolyte possesses a greater capability to suppress HER. After adding the SCL, the pH value increases from 5.06 to 5.56, which can enhance the corrosion resistance of the SCL/Zn(OTF)_2_ electrolyte (Fig. S8). As evidenced by the Tafel test results (Fig. S9), the SCL/Zn(OTF)_2_ electrolyte demonstrates a lower corrosion current density and a higher corrosion potential than the Zn(OTF)_2_ electrolyte [31]. Additionally, the surface morphology observations of zinc electrodes after being immersed in the SCL/Zn(OTF)_2_ electrolyte provide further evidence of the corrosion mitigation effect of the SCL/Zn(OTF)_2_ electrolyte (Fig. S10). Compared to Zn(OTF)_2_, no distinct characteristic peaks associated with the by-product, *e.g.,* Zn_x_(OTF)_y_(OH)_2x-y_·nH_2_O [32, 33], were observed on the Zn plate immersed in the SCL/Zn(OTF)_2_ electrolyte (Fig. S11). To further evaluate the interfacial stability, Zn//Zn symmetric batteries were tested galvanostatically (Fig. S12). In the SCL/Zn(OTF)_2_ electrolyte, the Zn anode’s interfacial impedance decreased progressively with cycling and stabilized by the 10th cycle. By contrast, in the Zn(OTF)_2_ electrolyte, impedance dropped sharply after the first cycle, then fluctuated erratically. This discrepancy likely stems from by-product accumulation and interfacial degradation due to irregular Zn deposition.

Molecular dynamics (MD) simulations were conducted to investigate the solvation structures of Zn^2+^ in the hybrid electrolyte. Figure 2b demonstrates that SCL effectively displaces H_2_O molecules within the primary solvation shell, thereby reconstructing the solvation structure of Zn^2+^. The activation energy (Ea) for the Zn interface in the SCL/Zn(OTF)_2_ electrolyte was calculated to be 27.0 kJ mol^−1^ (Fig. S13), markedly lower than the 34.8 kJ mol^−1^ observed in the Zn(OTF)_2_ electrolyte. This reduction in the de-solvation energy barrier suggests that the incorporation of SCL into the Zn^2+^ solvation sheath can significantly accelerate de-solvation kinetics by modulating the local environment around Zn^2+^. The influence of SCL on the Zn(OTF)_2_ electrolyte environment was confirmed by FTIR (Fig. 2c). The blue shift in both the O–H stretching vibration (3000–3700 cm^−1^) [34, 35] and the O–H bending vibration (1500–1700 cm^−1^) [36, 37] indicates reduced hydrogen bond interaction among H_2_O molecules, which will lead to a weakened water activity. Figure 2d presents the ^1^H nuclear magnetic resonance (NMR) spectra. With the addition of SCL, the ^1^H signal of H_2_O shifts to a lower field, suggesting the reduced electron density at the ^1^H position, which is induced by the disruption of the original hydrogen-bonding network. As shown in Fig. 2e, after the introduction of SCL, the strong hydrogen bond peak narrows markedly, while the medium and weak hydrogen bond peaks broaden, which indicates that free water with hydrogen bonds is significantly broken, reducing water activity.

The effects of SCL on the Zn(OTF)_2_ electrolyte environment were further validated through radial distribution functions (RDFs) and coordination numbers (N(r)). As illustrated in Fig. 2d, e, the primary peaks corresponding to the Zn–O pair from both SCL and H_2_O are observed at ~ 2 Å within the SCL/Zn(OTF)_2_ system. The coordination numbers for Zn–O (H_2_O) and Zn–O (SCL) are determined to be 5.71 and 0.04, respectively. This finding indicates that SCL successfully participates in the first solvation shell of Zn^2+^ ions [38]. The ab initio molecular dynamics (AIMD) simulation reveals the stable adsorption of SCL on the Zn surface, which can effectively inhibit water molecules from interacting with the Zn surface (Fig. S14).

### Influence of SCL on Zinc Deposition Behavior

**Fig. 3:**
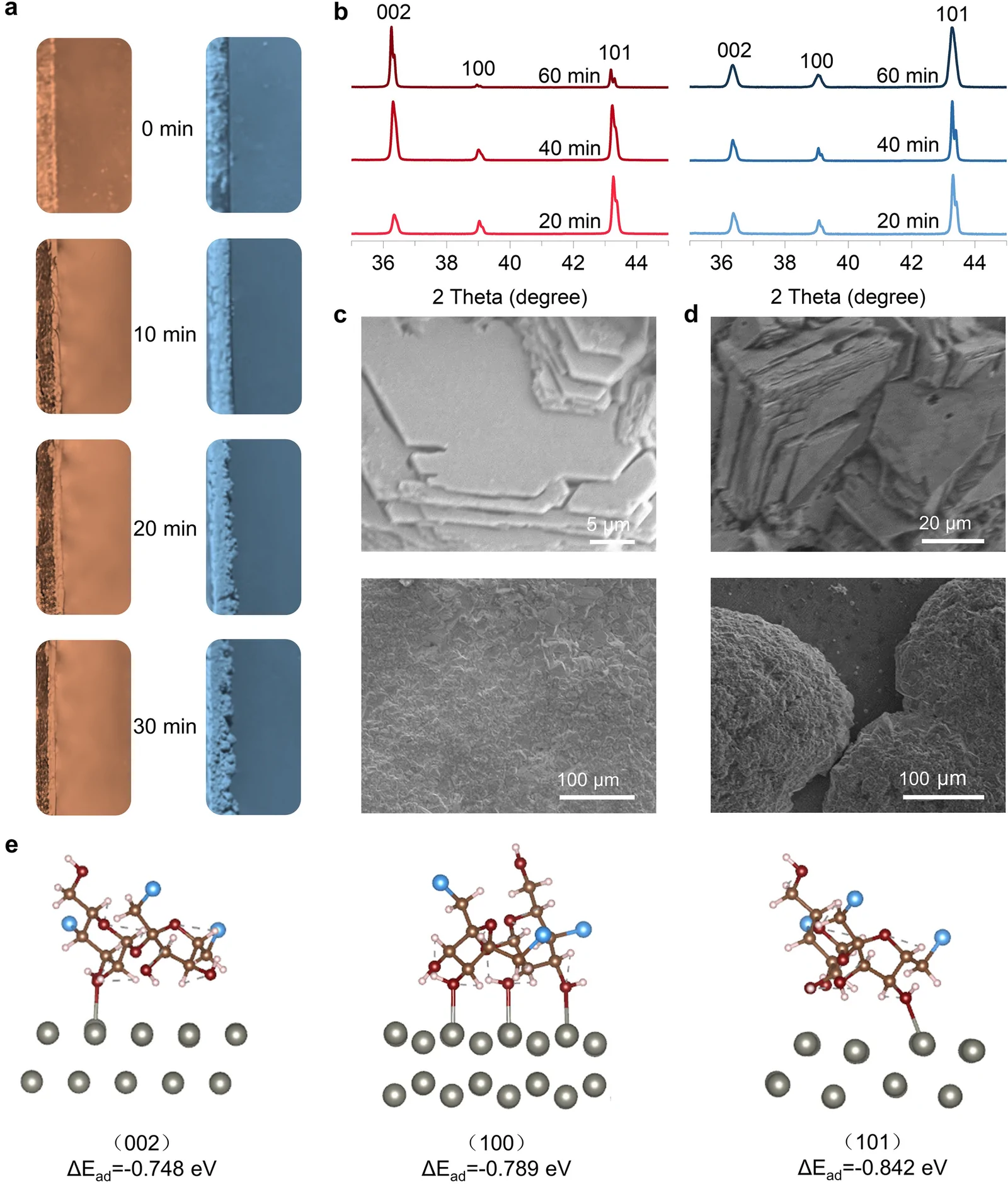
**a** In situ optical microscopic observations of Zn deposition in the SCL/Zn(OTF)_2_ (the left) and Zn(OTF)_2_ (the right) electrolytes with the current density of 30 mA cm^−2^. **b** XRD patterns of the fabricated Zn layer in SCL/Zn(OTF)_2_ (the left) and Zn(OTF)_2_ (the right) electrolyte systems with varying deposition times under 30 mA cm^−2^. SEM image of Zn deposits at 30 mA cm^−2^ in **c** SCL/Zn(OTF)_2_ and **d** Zn(OTF)_2_ electrolyte. **e** Zn ion adsorption energy on different crystal planes after SCL is adsorbed on the Zn slab

The actual plating process of Zn^2+^ was directly observed using in situ optical microscopy. In the Zn(OTF)_2_ electrolyte, ongoing the plating, prominent Zn dendrites appeared on the surface of the Zn electrode (Fig. 3a). In contrast, no dendrites were detected on the surface of the Zn electrode even after 30 min of plating in the SCL/Zn(OTF)_2_ electrolyte. To more accurately assess the influence of the SCL additive on the deposition behavior of Zn^2+^, the zinc layers deposited in different electrolytes were analyzed using XRD. The relative texture coefficient (RTC) of Zn deposition in the SCL/Zn(OTF)_2_ and Zn(OTF)_2_ electrolytes with the current density of 30 mA cm^−2^ is calculated using the following equation [39, 40]:

3 $${\mathrm{RCT}}_{{\left( {{\mathrm{HKL}}} \right)}} = \frac{{I_{{\left( {{\mathrm{HKL}}} \right)}} /I_{{0\left( {{\mathrm{HKL}}} \right)}} }}{{\Sigma \left[ {I_{{\left( {{\mathrm{HKL}}} \right)}} /I_{{0\left( {{\mathrm{HKL}}} \right)}} } \right]}} \times 100\%$$

The RTC value can be used to assess zinc deposition orientation and crystal growth behavior during electroplating. As the RTC value increases, electroplated Zn tends to align horizontally. On the contrary, as the RTC values decreases, the deposited Zn tends to be irregularly inclined Zn dendrites. As shown in Fig. 3b, Zn deposited with SCL shows a continuous increase in the peak intensity of Zn (002) crystal facet during the plating process, the RTC value increased from 20 to 47 and 76 after 20, 40 and 60 min of deposition (Fig. S15), respectively. When deposition in Zn(OTF)_2_ electrolyte with the same condition, the RTC value exhibited minimal variation during the electroplating process. At other current densities, the addition of SCL shows the same influence on the Zn depositing behavior (Fig. S16). The preferential (002) texture favors the uniform and consistent growth of Zn. As shown by the SEM observation (Fig. 3c, d), the Zn anode deposited in the Zn(OTF)_2_ electrolyte exhibits nonuniform deposition at 20 mA cm^−2^—20 mAh cm^−2^. In the SCL/Zn(OTF)_2_ electrolyte, the deposited Zn shows a clear hexagonal shape and is closely packed, consistent with the optical observation. As the current density increases, the deposited zinc shows a more uniform and compact surface (Fig. S17). To further investigate the stability of the influence exerted by SCL during cycling, we conducted SEM characterization to examine the morphology of Zn anodes. After 10 cycles, the Zn in the blank group displays uneven growth accompanied by the gradual emergence of dendrites. In contrast, the Zn in the SCL/Zn(OTF)_2_ group exhibits uniform growth characterized by numerous horizontally aligned Zn plates on its surface (Figs. S18 and S19).

Figure 3e presents the side views of Zn atom adsorption configurations on the (100), (101), and (002) crystallographic planes, along with the adsorption energies, respectively. After adsorbing SCL onto three different Zn facets, the adsorption energy of Zn atoms on Zn (002)-SCL shows the lowest value (absolute value). According to Bravais’ law, the orientation of crystal planes is determined by the ion deposition rates on various planes. The plane with the slowest growth rate tends to become the dominant exposed facet [41]. When Zn(002), (100), and (101) coexist, newly electroplated zinc tends to nucleate and grow preferentially on the (100) and (101) facets due to their higher adsorption energy. The selective growth leads to the formation of a flat, preferred (002) texture.

**Fig. 4:**
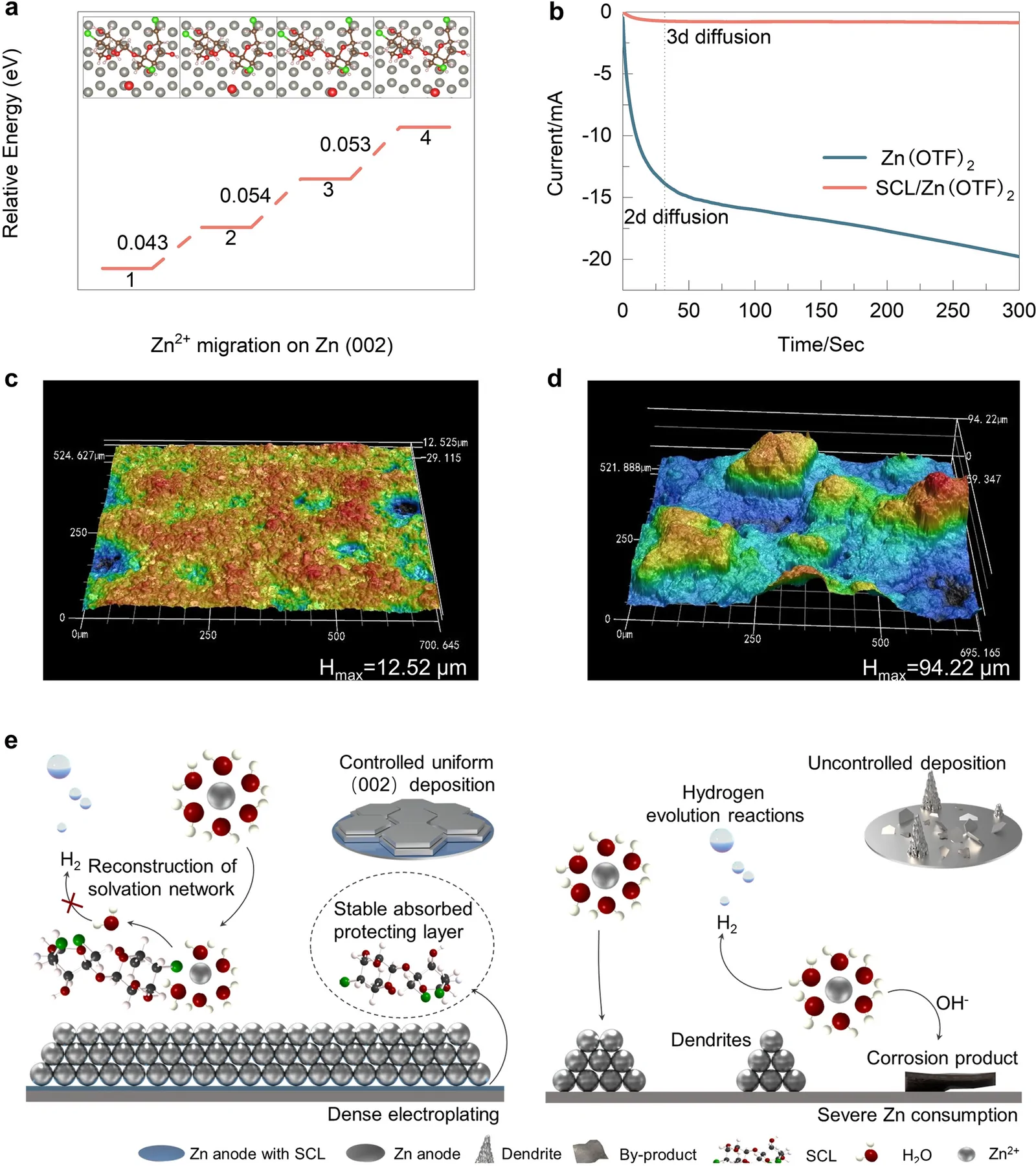
**a** Zn^2+^migration energy on Zn (100) after SCL adsorption, insets show the calculated migration positions. **b** CA profiles in different electrolytes under a fixed overpotential of − 150 mV. The 3D surface topographies of the deposited Zn electrodes surface in **c** SCL/Zn(OTF)_2_ electrolyte and **d** Zn(OTF)_2_ electrolyte at 30 mA cm^−2^. **e** Schematic illustration of Zn plating behavior with and without SCL additive

The Zn^2+^ deposition rate discrepancies across diverse crystal planes are further interpreted via Zn^2+^ migration energy. On the SCL-adsorbed Zn crystal surface, the migration energy of Zn^2+^ ions at different sites is lower on the Zn(002) plane compared to the Zn(100) and (101) planes (Figs. 4a and S20), which implies a more rapid migration of Zn ions on the Zn(002) plane Consequently, Zn ions can easily diffuse along the Zn(100) and (101) directions to other planes, facilitating the exposure of the layered Zn(002) texture. This phenomenon can be deemed as homoepitaxial growth [42]. The nucleation and deposition behavior of the Zn electrode were investigated using the chronoamperometry (CA) technique by analyzing the current responses at a fixed overpotential of − 150 mV (Fig. 4b). The deposition in Zn(OTF)_2_ electrolyte exhibits a continuously increasing current over 300 s, suggesting uncontrolled 2D diffusion of Zn^2+^ ions [43, 44]. This phenomenon can be attributed to the continuous increase in electrode surface area caused by the formation of the “tip effect” [45]. The elevated nucleation overpotential observed in the CV curves (Fig. S21) indicates that the SCL additive enhances the initial nucleation barrier of the Zn anode by promoting its adsorption, leading to the formation of finer nuclei and a decelerated nucleation process within the SCL/Zn(OTF)_2_ system, which in turn enables the development of a dense deposition layer. In systems that incorporate SCL, the trend of increasing current is relatively moderate, demonstrating that the SCL can suppress Zn dendrite growth. The 3D confocal laser scanning microscopy (CLSM) images in Fig. 4c, d visually show the surface state of the deposited Zn. The addition of SCL leads to a smooth surface. While in the Zn(OTF)_2_ electrolyte, the deposited Zn exhibits significant inhomogeneity and features numerous sharp protrusions.

Based on the experimental results and computational analyses, it can be concluded that the introduction of SCL can effectively direct the uniform plating of Zn^2+^. As illustrated by Fig. 4e, the SCL additive can not only disrupt the solvation structure around zinc ions and reduce water activity but also preferentially adsorb onto the Zn(002) crystal surface, inducing stable zinc deposition and slowing down both nucleation and diffusion of Zn^2+^, promoting enhanced exposure of the Zn(002) crystal facet. Meanwhile, SCL adsorbed on the zinc anode surface serves to prevent direct contact between free water and zinc foil, acting as a protective barrier that further inhibits HER and corrosion.

### Electrochemical Performance of Batteries with SCL Additive

**Fig. 5:**
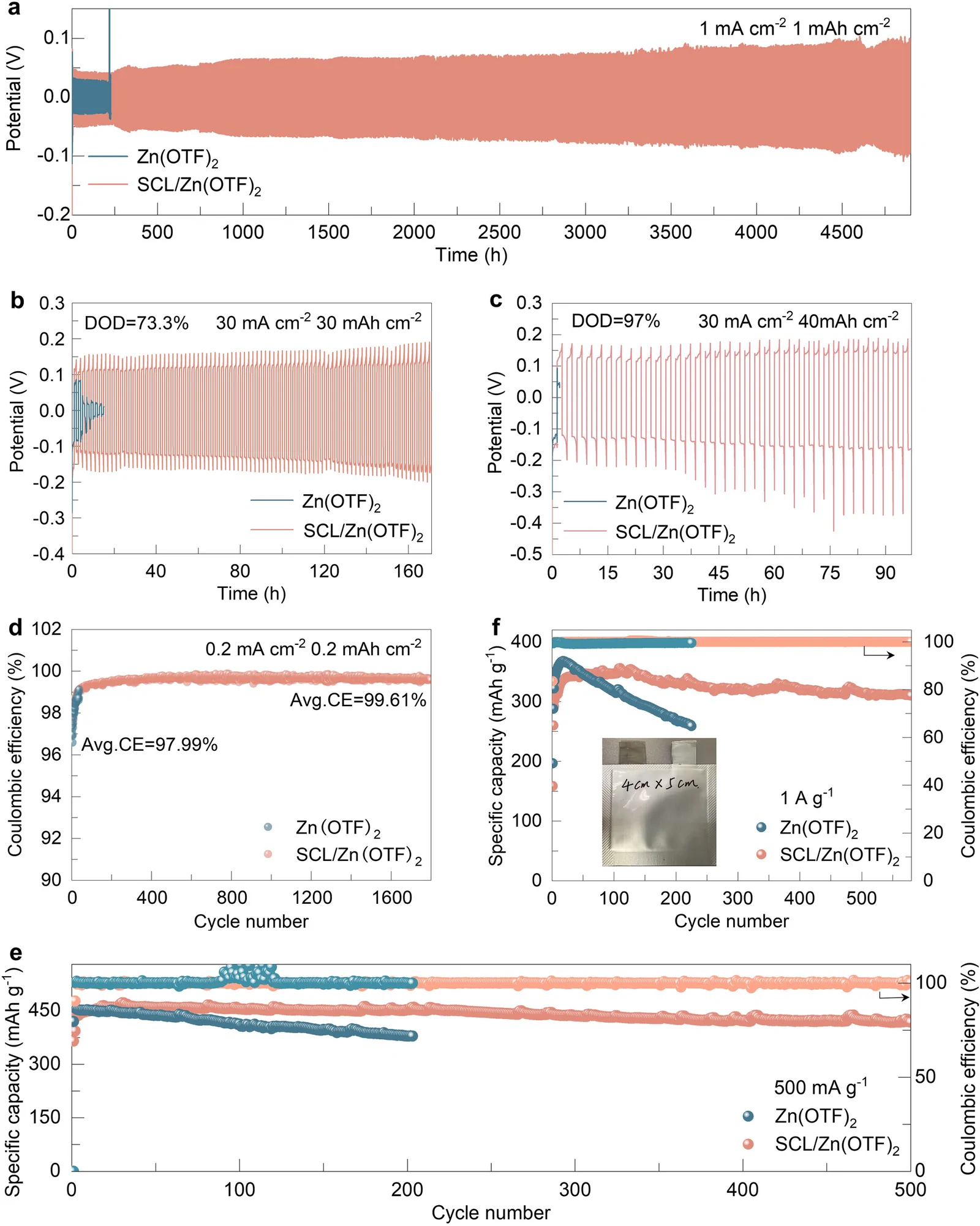
**a** Galvanostatic cycling of Zn//Zn battery in bare Zn(OTF)_2_ and SCL/Zn(OTF)_2_ electrolyte at 1 mA cm^−2^, 1 mAh cm^−2^. **b** Galvanostatic cycling of Zn//Zn battery with a limited Zn supply (DOD_Zn_ = 73.3%) in SCL/Zn(OTF)_2_ electrolyte at 30 mA cm^−2^, 30 mAh cm^−2^. **c** Galvanostatic cycling of Zn//Zn battery with a limited Zn supply (DOD_Zn_ = 97%) in SCL/Zn(OTF)_2_ electrolyte at 30 mA cm^−2^, 40 mAh cm^−2^. **d** CE of Zn//Cu half batteries with various electrolytes at 0.2 mA cm^−2^, 0.2 mAh cm^−2^. **e** Long-term cycling performance and corresponding CEs of Zn//NH_4_V_4_O_10_ full batteries at 500 mA g^−1^. **f** Electrochemical performance of NH_4_V_4_O_10_//Zn pouch cells operated in bare Zn(OTF)_2_ and SCL/Zn(OTF)_2_ electrolyte at 1 A g^−1^ (mass loading = 1.5 mg cm^−2^, N/P = 39.2, E/C = 33.5 *μ*L mAh.^−1^)

The electrochemical performance of Zn//Zn and Zn//Cu batteries with and without SCL was tested. At 1 mA cm^−2^–1 mAh cm^−2^ (Figs. 5a and S22), the battery with SCL/Zn(OTF)_2_ shows much longer cycling time (4900 h) compared with that in Zn(OTF)_2_ (224 h). Even at a higher current density and areal capacity of 30 mA cm^−2^ and 30 mAh cm^−2^ (DOD = 73.3%), the battery still exhibits stable cycling performance for 171 h, accompanied by steady potential profiles (Fig. 5b). Practical applications require the evaluation of Zn electrodes’ endurance capabilities under the concurrent challenges of high current densities, high areal capacities, and deep depths of discharge. With a much higher DOD of 97.7% (30 mA cm^−2^–40 mAh cm^−2^), a lifespan of 98 h can still be achieved for the Zn//Zn batteries (Fig. 5c), which is superior to many previously reported works (Table S1). Figure S23 shows the digital photos of Zn//Zn symmetric battery after cycling. Clearly, the battery with the SCL/Zn(OTF)_2_ electrolyte shows reduced volume expansion than that with Zn(OTF)_2_, indicating a suppressed HER [46]. The rate performance (Fig. S24) further demonstrates that the batteries can stably operate using the SCL/Zn(OTF)_2_ electrolyte across a broad current density range, from 1 to 30 mA cm^−2^. Figure. S25 shows the coulombic efficiency of Zn//Cu battery at 0.5 mA cm^−2^ and 0.5 mAh cm^−2^. With the SCL/Zn(OTF)_2_ electrolyte, the Zn//Cu battery exhibits excellent reversibility for 1000 cycles and the average CE is as high as 99.84%. Currently, reported CE values are typically obtained at current densities of 0.5 mA cm^−2^ or higher, as elevated current densities tend to obscure the effects of the HER, which will lead to a high CE value [47]. Here, to show the superiority of the SCL/Zn(OTF)_2_ electrolyte, at a very low current density of 0.2 mA cm^−2^ and low area capacity of 0.2 mAh cm^−2^, we tested the coulombic efficiency of Zn//Cu batteries. As shown in Figs. 4d and S26, the Zn//Cu battery with the SCL/Zn(OTF)_2_ electrolyte still shows a high CE of 99.61% and maintains a long lifespan of over 4000 h, superior to that with Zn(OTF)_2_ electrolyte and previously reported works (Table S2), indicating the high reversibility of Zn plating/stripping induced by the addition of SCL.

Next, Zn//NH_4_V_4_O_10_ full batteries were assembled to investigate the feasibility of SCL/Zn(OTF)_2_ electrolyte in practical battery systems. The CV profiles of the Zn///NH_4_V_4_O_10_ batteries employing the SCL/Zn(OTF)_2_ and Zn(OTF)_2_ electrolytes indicate that the incorporation of SCL has negligible influence on the redox behavior of the NH_4_V_4_O_10_ cathode (Fig. S27). However, as shown in Fig. 5e, at 500 mA g^−1^, the battery with SCL/Zn(OTF)_2_ electrolyte demonstrates a long lifespan, achieving a capacity retention of 90.7% (420 mAh g^−1^ after 500 cycles). In contrast, the battery without SCL becomes inoperative after only 203 cycles. Figure. S28 presents the corresponding voltage profiles at specific cycle numbers under a current density of 500 mA g^−1^, illustrating enhanced capacity retention with SCL/Zn(OTF)_2_ electrolyte. Furthermore, the cycling performance of full battery at 5 A g^−1^ shows 84.9% capacity retention after 4000 cycles (Figs. S29 and S30). Figure. S31 presents the rate performance of Zn//NH_4_V_4_O_10_ full batteries, indicating that the battery incorporating SCL exhibits superior rate performance. To further validate its potential application, we fabricated a Zn//NH_4_V_4_O_10_ pouch cell utilizing an electrolyte augmented with SCL for cyclic evaluation. The pouch cell was assembled with single anode and cathode components within dimensions of 20 cm^2^ (4 cm × 5 cm). With SCL/Zn(OTF)_2_ electrolyte, the pouch cell maintains a reversible capacity of 311 mAh g^−1^ over 580 cycles at a current density of 1 A g^−1^ (Fig. 5f), superior to that using the Zn(OTF)_2_ electrolyte.

## Conclusion

In conclusion, an electrolyte engineering strategy was proposed to address critical issues related to dendrite growth and by-product generation, aiming to enhance the long-term stability of zinc-ion batteries. After introducing sucralose (SCL) into the electrolyte, the as-formed SCL adsorption layer on the Zn surface leads to the exposure of (002) facet which promotes horizontal deposition of Zn and can effectively restrict side reactions. Employing the SCL/Zn(OTF)_2_ electrolyte, the Zn//Zn symmetric battery can stably run for 4900 h at 1 mA cm^−2^–1 mAh cm ^−2^ and 171 h at 30 mA cm^−2^–30 mAh cm^−2^ (depth of discharge, DOD = 73.3%). Even at an ultrahigh DOD of 97.7% (30 mA cm^−2^–40 mAh cm^−2^), the Zn//Zn symmetric battery can maintain for 98 h. At a very low current density of 0.2 mA cm ^−2^ and small area capacity of 0.2 mAh cm^−2^, the Zn//Cu half battery shows a high average CE of 99.61% and a long lifespan over 4000 h, indicating high reveribility and stability of the Zn anode enabled by the SCL. With the SCL/Zn(OTF)_2_ electrolyte, the Zn//NH_4_V_4_O_10_ full battery delivers 420 mAh g^−1^ at 500 mA g^−1^ after 500 cycles, retaining 90.7% of its capacity. Additionally, the pouch cell maintains a reversible capacity of 311 mAh g^−1^ over 580 cycles when operated at 1 A g^−1^. This work provides a practical and efficient electrolyte additive to drive the practical development of AZIBs.

## Supplementary Information

Below is the link to the electronic supplementary material.

Supplementary file1 (DOCX 20164 KB)
