# Supplementary material for: Exposing Zn(002) Texture with Sucralose Additive for Stable and Dendrite-Free Aqueous Zinc-Ion Batteries
Source: Nanomicro Lett. 2026 Jan 9;18:107. doi: 10.1007/s40820-025-01954-3 (PMC12783496; doi:10.1007/s40820-025-01954-3)
Supplement: Supplementary file 1 — Supplementary file1 (DOCX 20164 KB) [file 40820_2025_1954_MOESM1_ESM.docx]

# Supporting Information for

**Exposing Zn(002) Texture with Sucralose Additive for Stable and Dendrite-Free Aqueous Zinc-Ion Batteries**

Feiyu Tao^1,4^, Yingke Ren^2^, Li’e Mo^3^, Yifan Wang^3^, Yang Huang^3^, Hong Zhang^5^, Chengwu Shi^1^, Zhaoqian Li^1,3*^, Jiaqin Liu^1,6^, Lei Chen^4,*^, Linhua Hu^3,*^, Yucheng Wu^1,*^

^1^School of New Energy Engineering, Hefei Institute of Technology, Hefei, Anhui, 238076, P. R. China

^2^College of Science, Hebei University of Science and Technology, Shijiazhuang, Hebei 050018, P. R. China

^3^Key Laboratory of Photovoltaic and Energy Conservation Materials, CAS, Institute of Solid State Physics, Hefei Institutes of Physical Science, Chinese Academy of Sciences, Hefei, Anhui 230031, P. R. China

^4^School of Environmental and Chemical Engineering, Jiangsu University of Science and Technology, Zhenjiang 212003, P. R. China

^5^Hebei Computational Optical Imaging and Photoelectric Detection Technology Innovation Center, Hebei International Joint Research Center for Computational Optical Imaging and Intelligent Sensing, School of Mathematics and Physics Science and Engineering, Hebei University of Engineering, Handan, Hebei 056038, P. R. China

^6^School of Chemistry, Beijing University of Chemical Technology, Beijing 100029, P. R. China

*Corresponding authors. E-mail: [zqli@hfit.edu.cn](mailto:zqli@hfit.edu.cn) (Zhaoqian Li); [chenlei@just.edu.cn](mailto:chenlei@just.edu.cn) (Lei Chen); [lhhu@rntek.cas.cn](mailto:lhhu@rntek.cas.cn) (Linhua Hu); [ycwu@hfut.edu.cn](mailto:ycwu@hfut.edu.cn) (Yucheng Wu)

**Supplementary Figures and Tables**


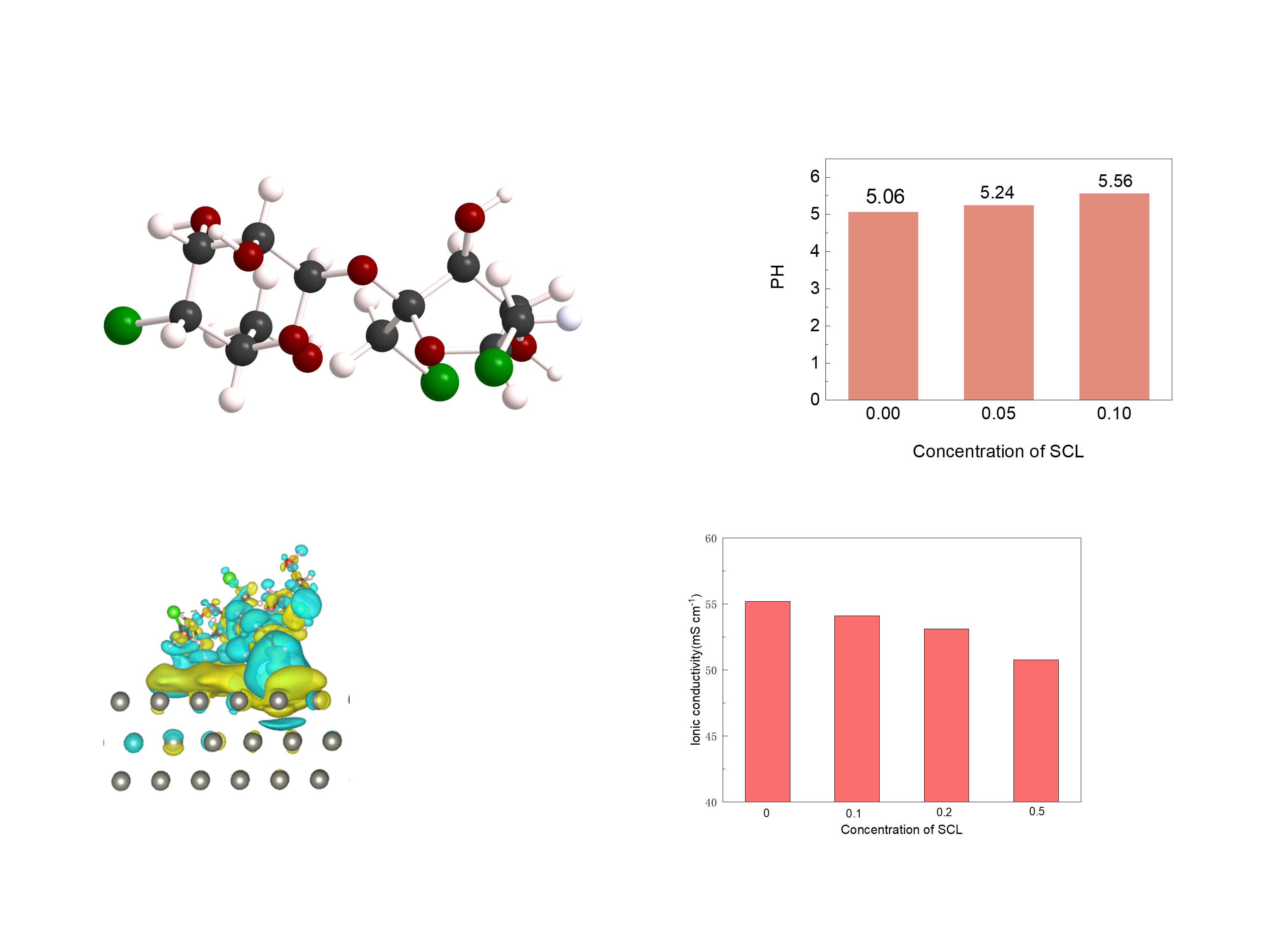


**Fig. S1** The molecule structure of SCL


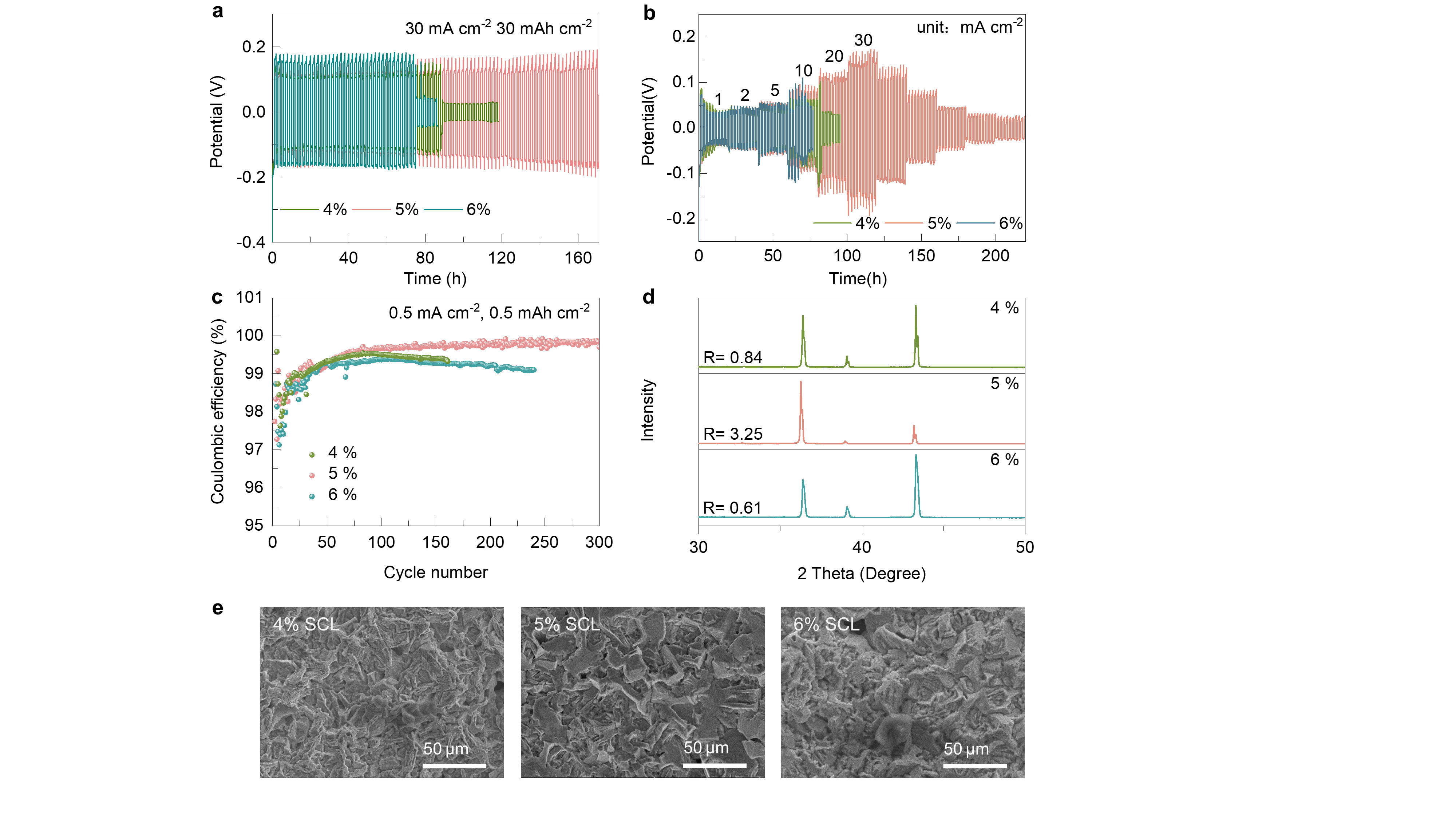


**Fig. S2 a** The voltage profiles of Zn//Zn batteries with different electrolytes at 30 mA cm^-2^ and 30mAh cm^-2^. **b** Rate cycling performance comparison of Zn//Zn symmetric cells under different current densities and areal capacities of 1 mA cm^-2^/1 mAh cm^-2^, 2 mA cm^-2^/2 mAh cm^-2^, 5 mA cm^-2^/5 mAh cm^-2^, 10 mA cm^-2^/10 mAh cm^-2^, 20 mA cm^-2^/20 mAh cm^-2^ and 30 mA cm^-2^/30 mAh cm^-2^. **c** CEs of Zn plating/stripping on Cu foil in SCL/ Zn(OTF)_2_ electrolyte at 0.5 mA cm^-2^ with the capacity of 0.5 mAh cm^-2^. **d** The XRD spectra of the deposited Zn plates in different electrolytes. **e** SEM image of the deposited Zn at a constant current density of 30 mA cm^-2^ in different electrolytes

The Zn//Zn symmetric battery results demonstrate that the electrolyte containing 5 wt% SCL exhibits the most extended cycling lifespan and exceptional rate performance, whereas other electrolytes suffer from short circuits after only a few cycles (Figs. S4a, S4b). As depicted in Fig. S4c, the 5 wt% SCL-containing Zn//Cu battery achieves the highest Coulombic efficiency. XRD analysis of Zn deposits formed at 30 mA cm^-2^ shows that the (002) diffraction peak is most intense in the 5 wt% SCL electrolyte, indicative of a preferential (002) orientation during Zn deposition. Furthermore, SEM observations (Fig. S4e) reveal that Zn deposited in the 5 wt% SCL-containing electrolyte exhibits a discernibly flat and layered morphology, contrasting sharply with the noticeably non-uniform and disordered structures observed in other tested electrolytes.


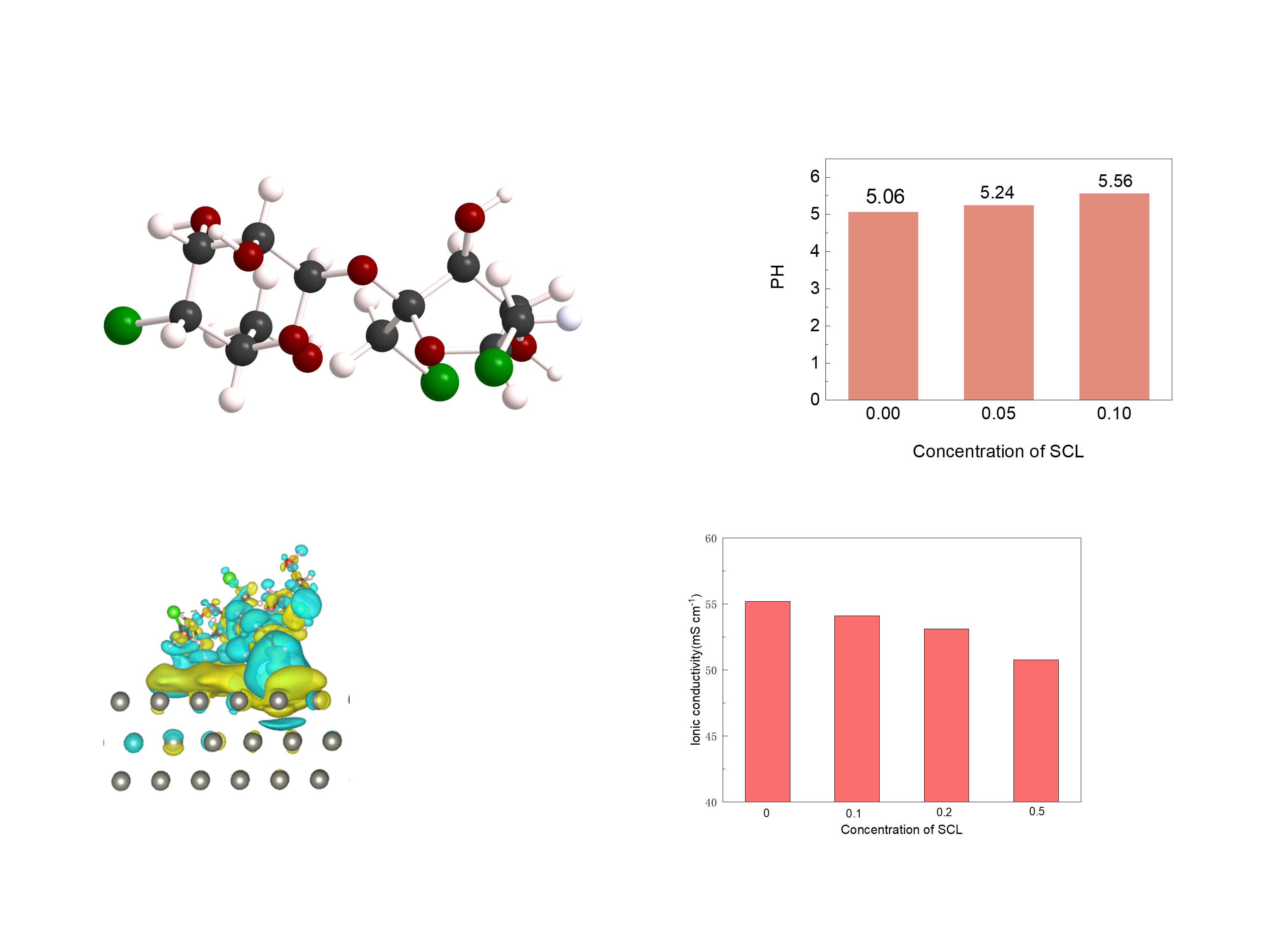


**Fig. S3** The charge density difference of SCL adsorbed on the Zn (002)


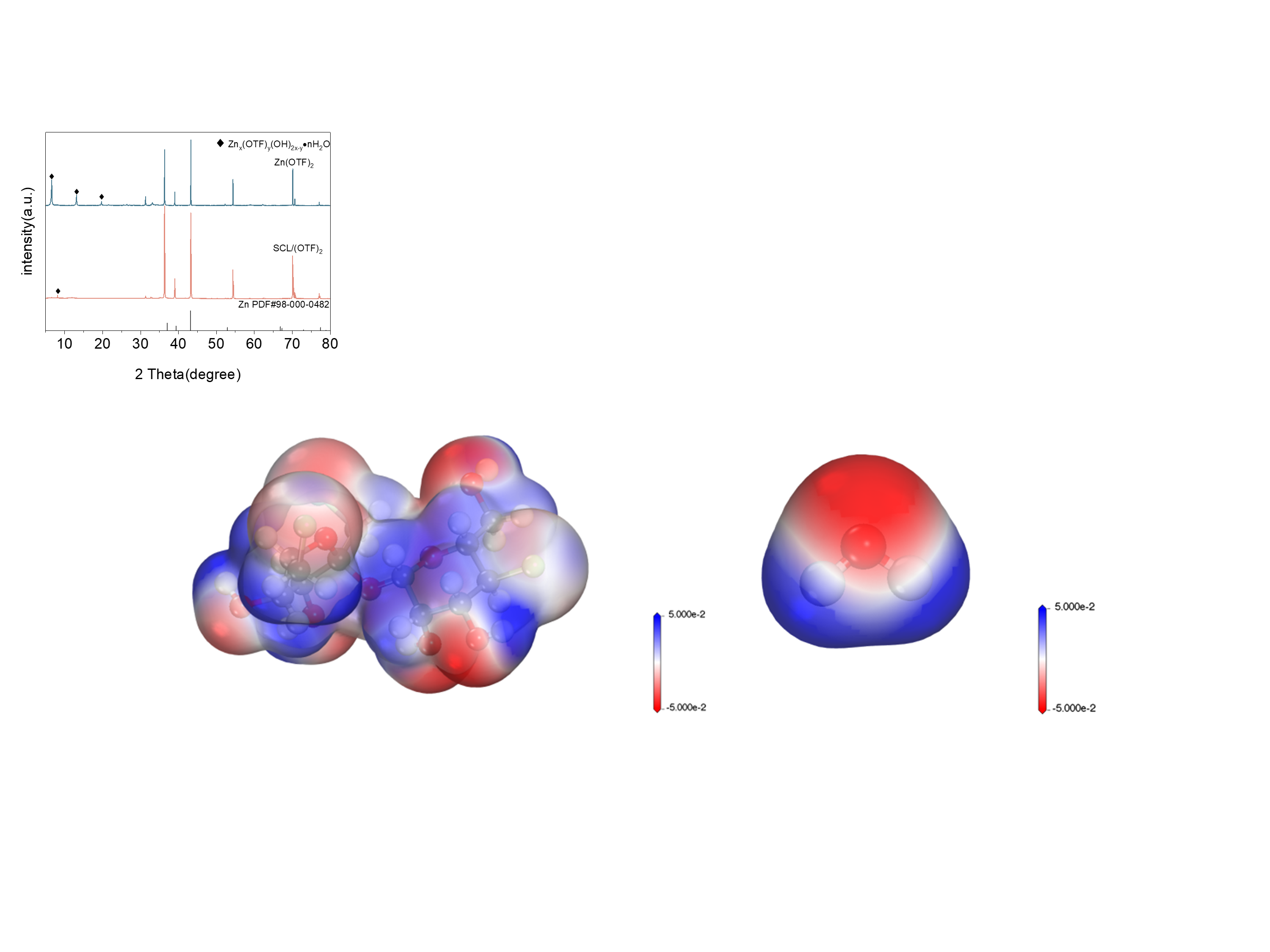


**Fig. S4** Electrostatic potential (ESP) mapping of SCL molecule


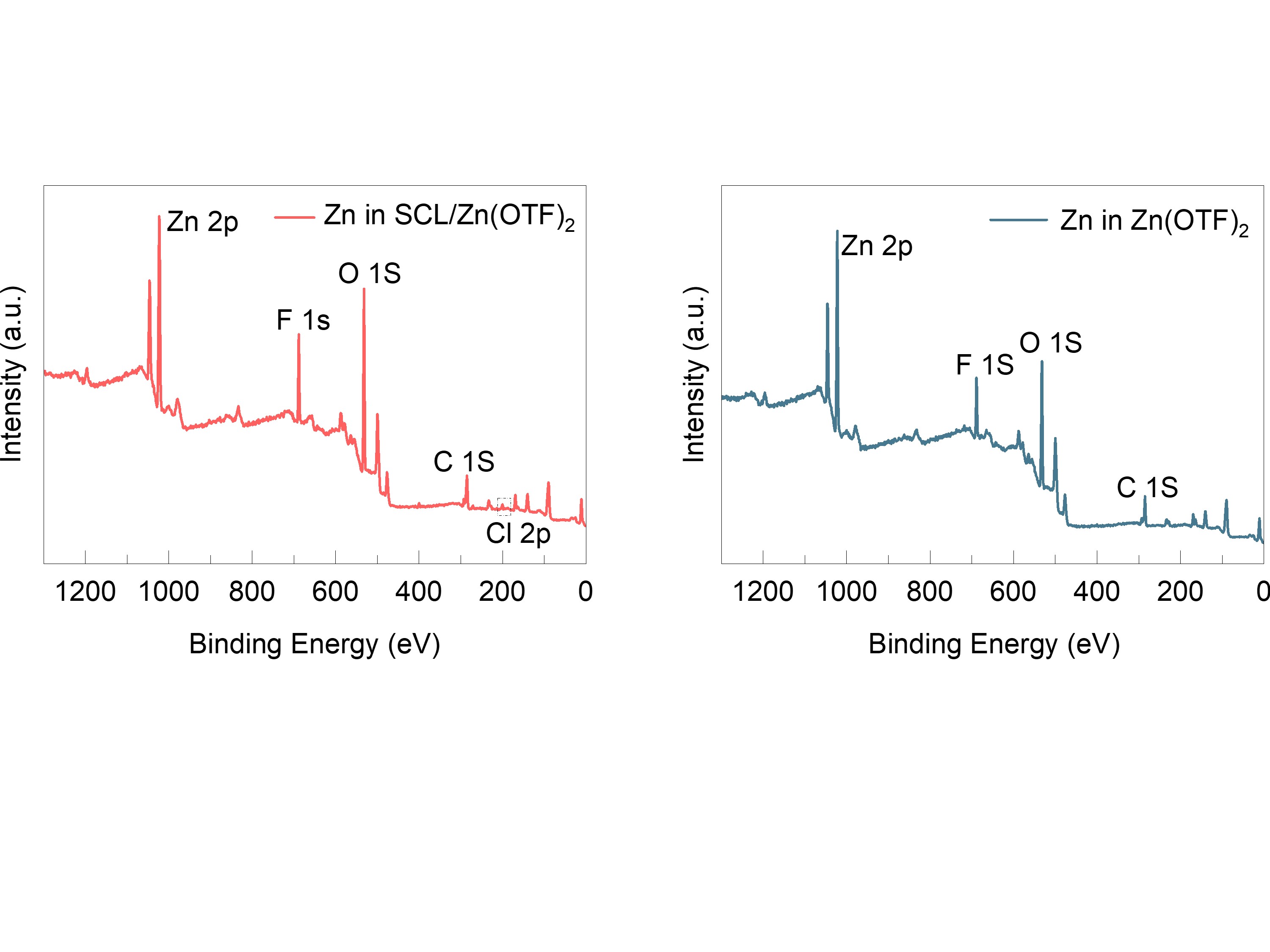


**Fig. S5** XPS spectra for Zn foil after soaking in electrolytes with/without the SCL additive for 24 h


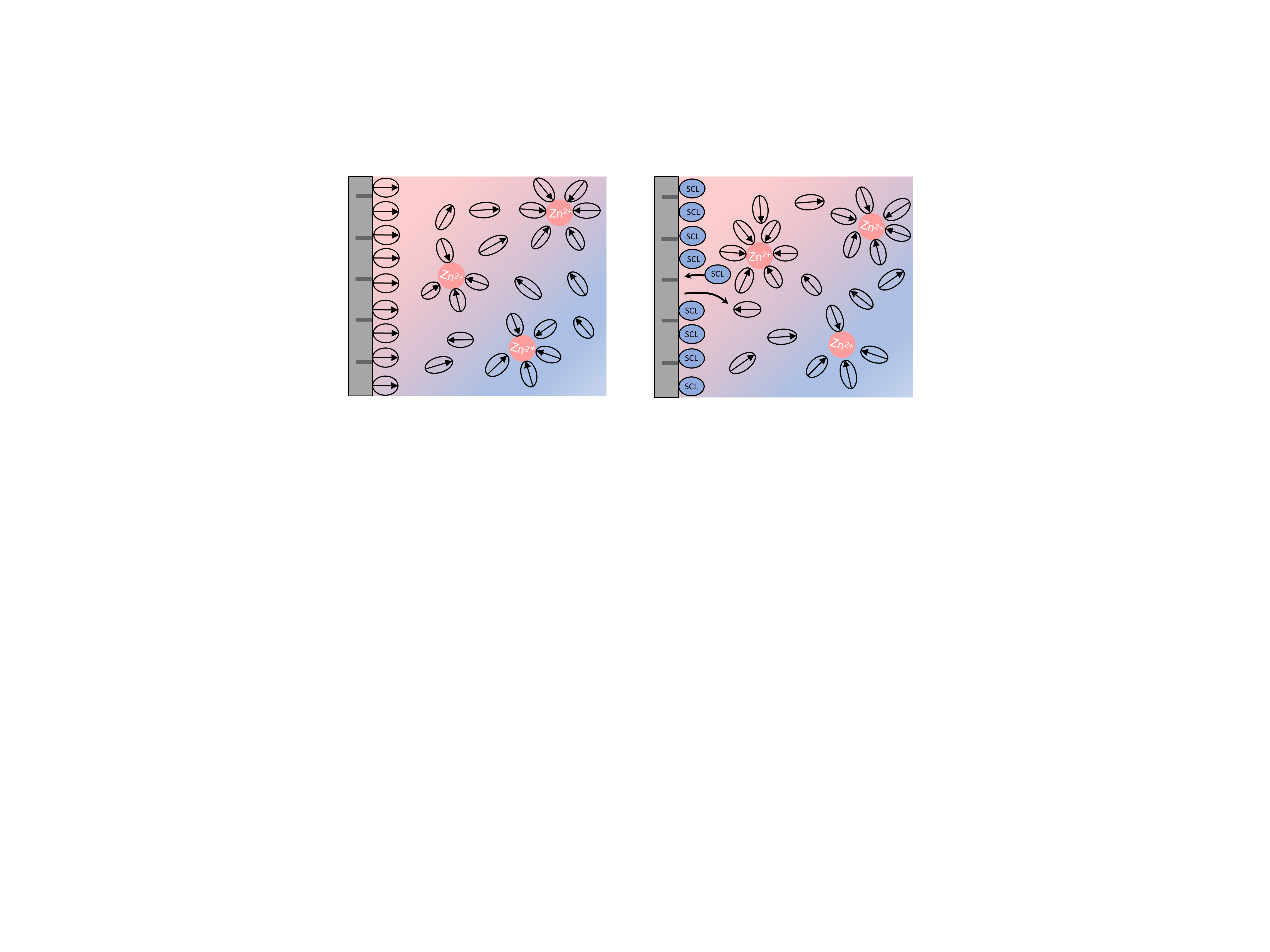


**Fig. S6** The schematic descriptions of the interface structure: after (the right) and before (the left) introducing SCL


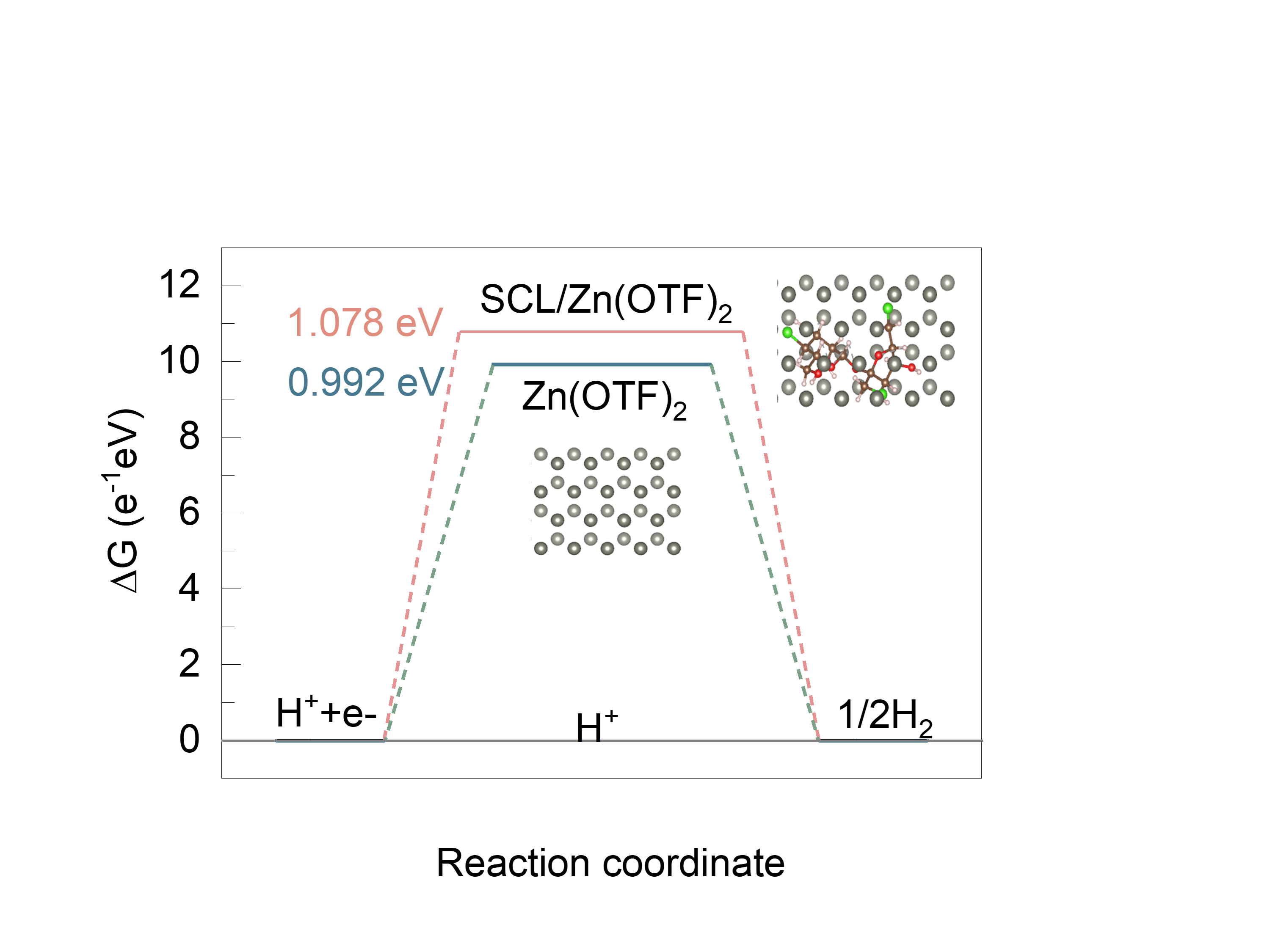


**Fig. S7** The hydrogen barrier of pure Zn(OTF)_2_ and SCL/Zn(OTF)_2_ electrolytes


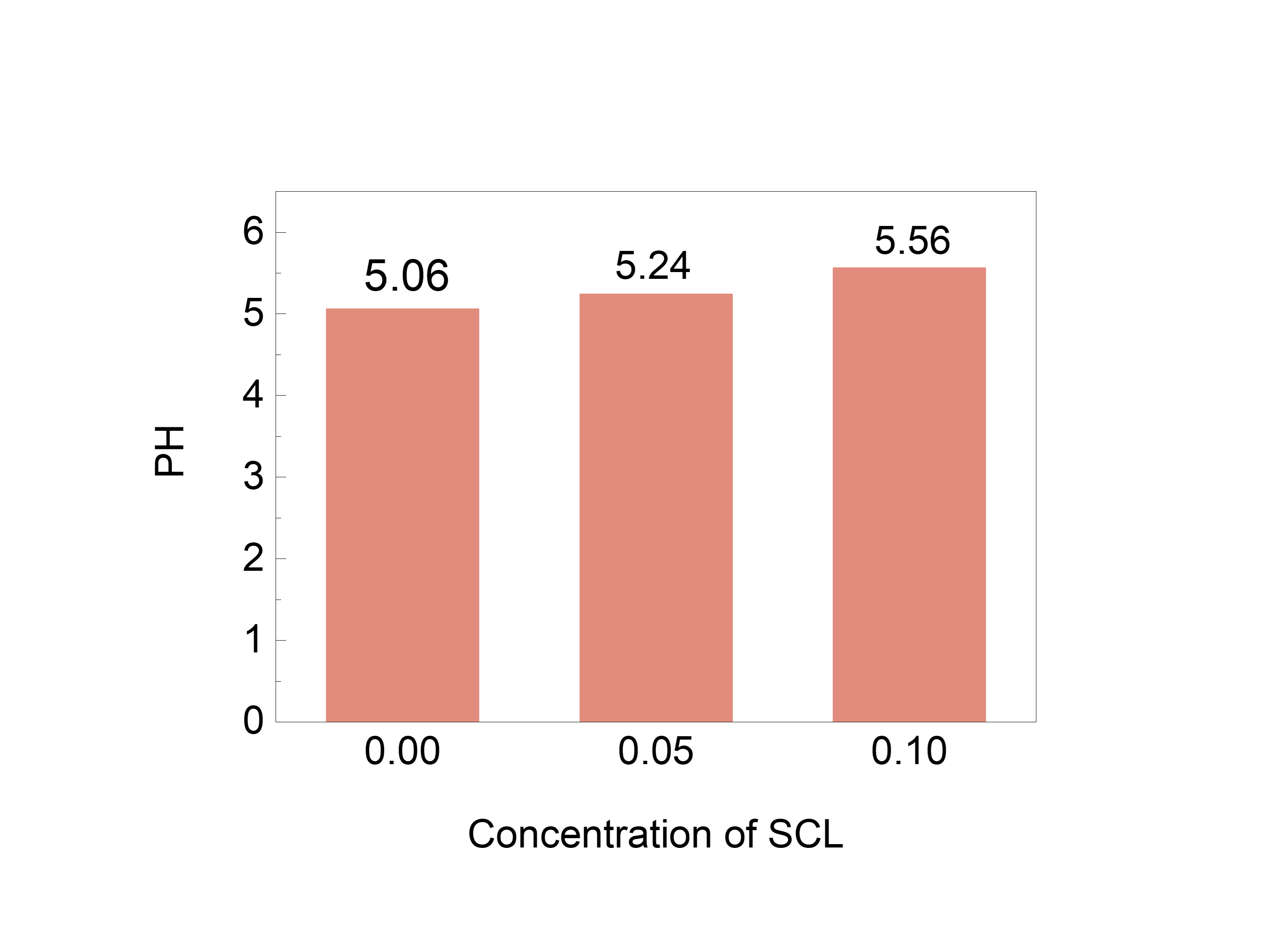


**Fig. S8** pH measurement of SCL/Zn(OTF)_2_ solution


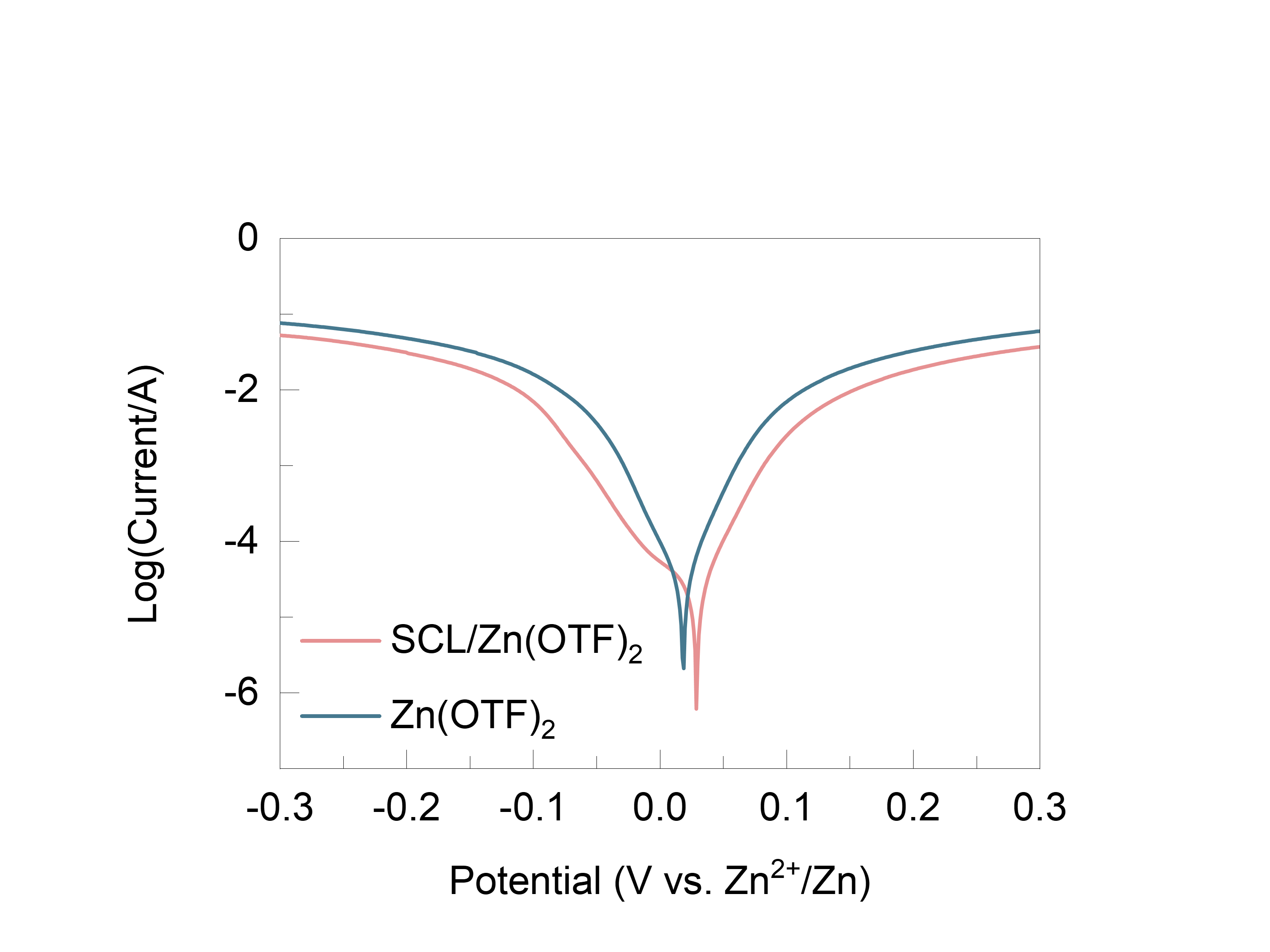


**Fig. S9** The Tafel plots in different electrolytes


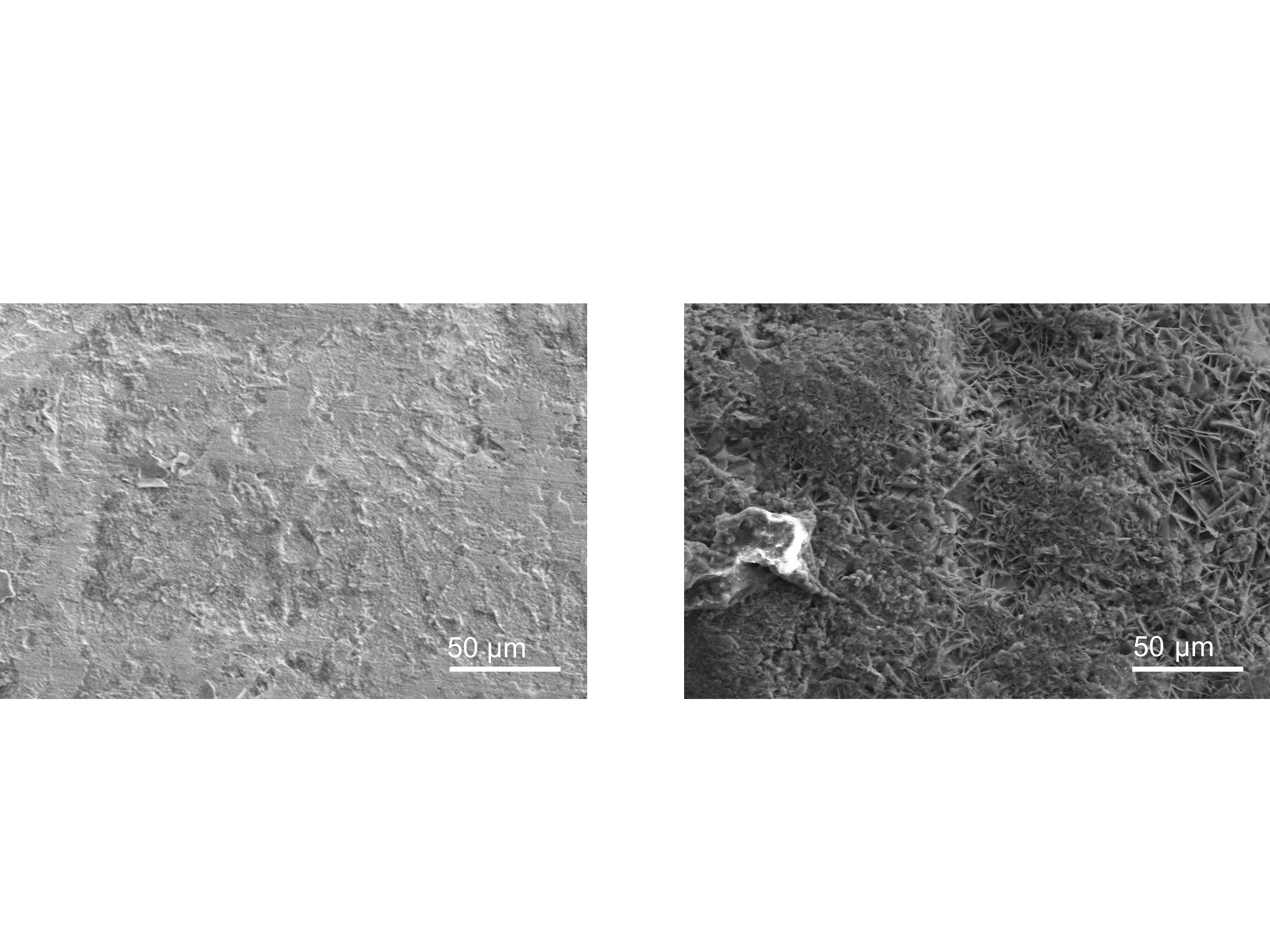


**Fig. S10** The SEM images of soaked Zn plated surface in SCL/Zn(OTF)_2_ (the left) and Zn(OTF)_2_ (the right)


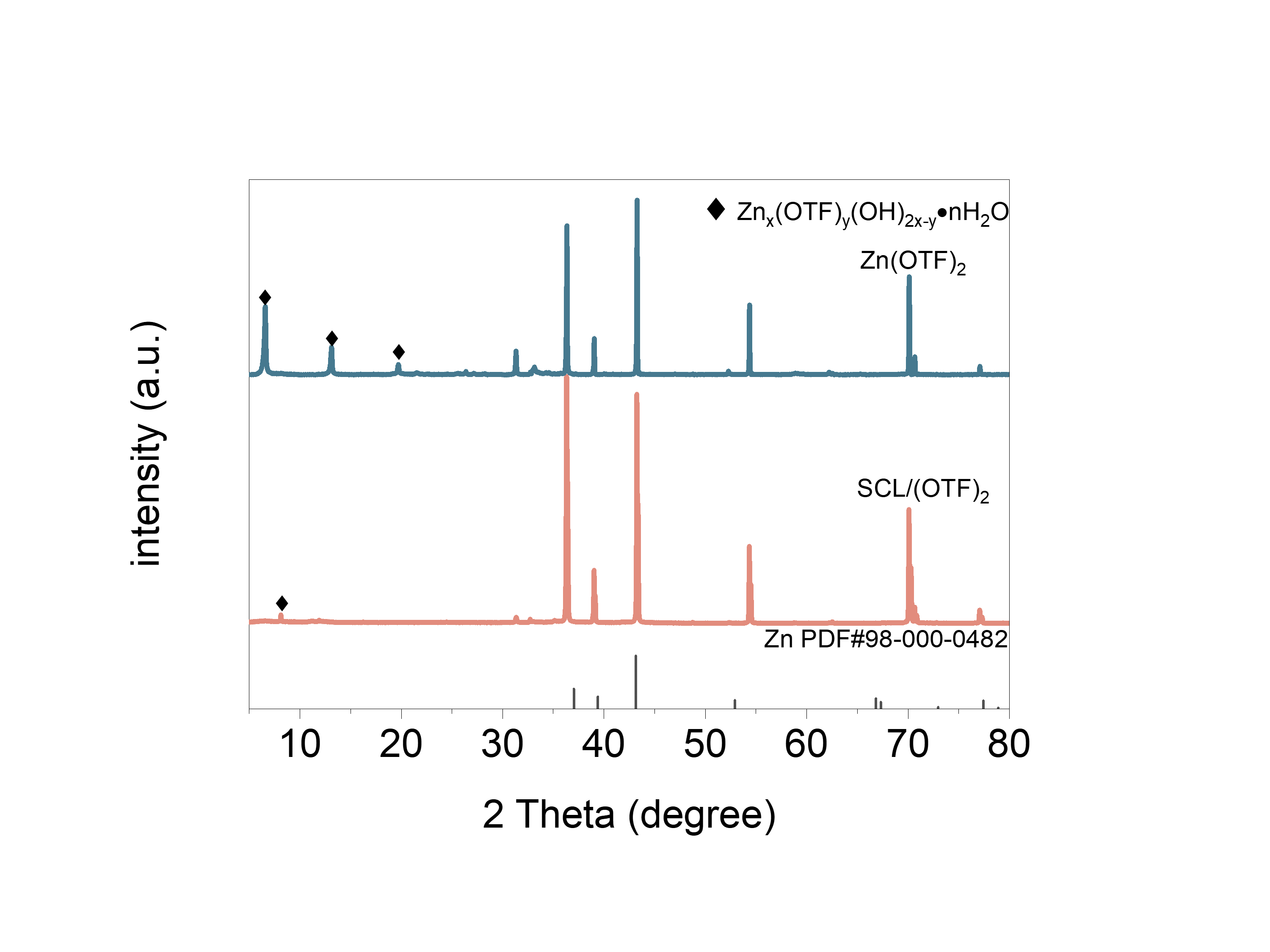


**Fig. S11** The XRD curves of Zn anode immersed in SCL/Zn(OTF)_2_ and Zn(OTF)_2_ electrolyte


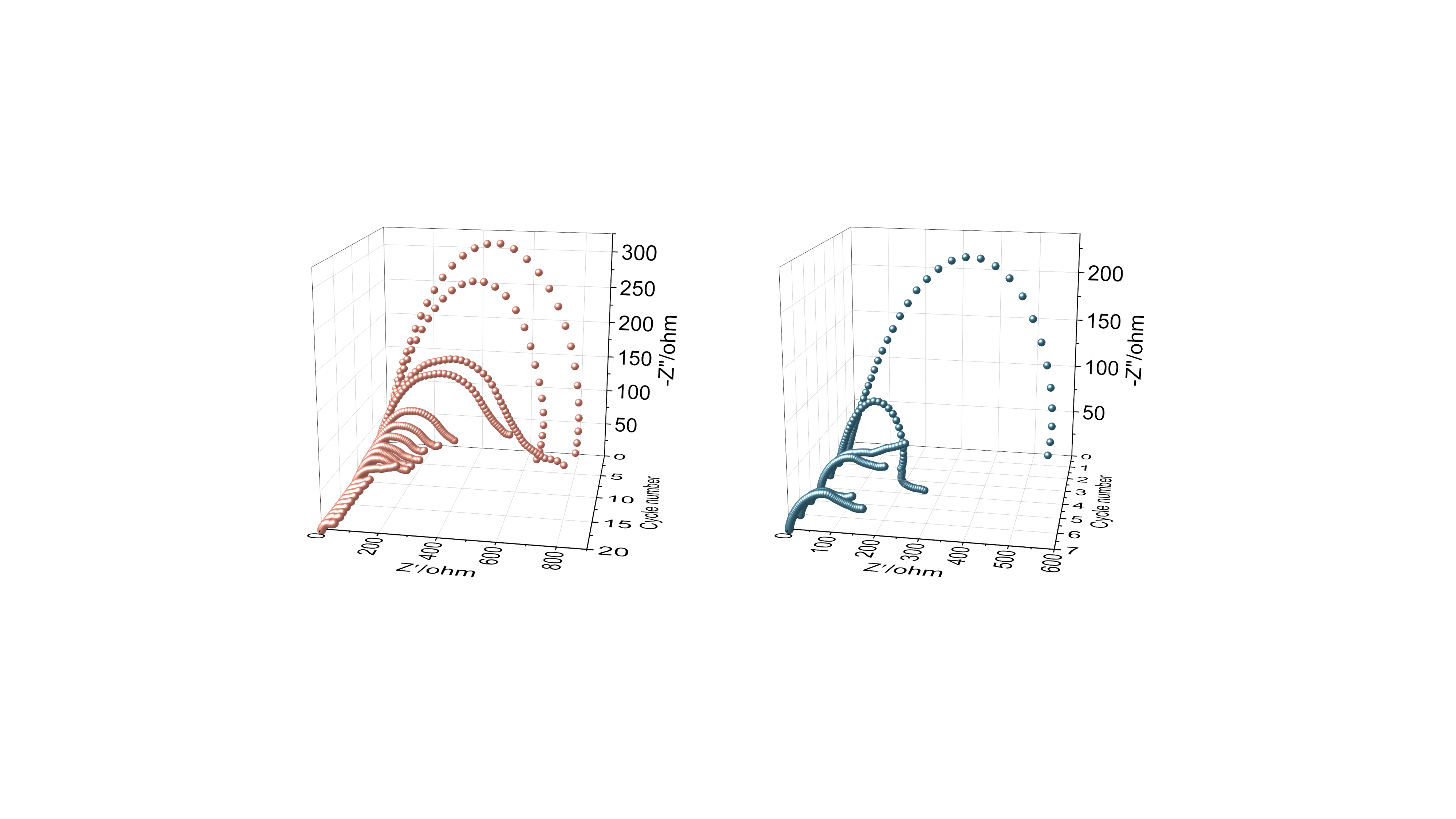


**Fig. S12** EIS of Zn//Zn symmetric cells with SCL/Zn(OTF)_2_ (left) and Zn(OTF)_2_ (right) electrolytes after different cycles


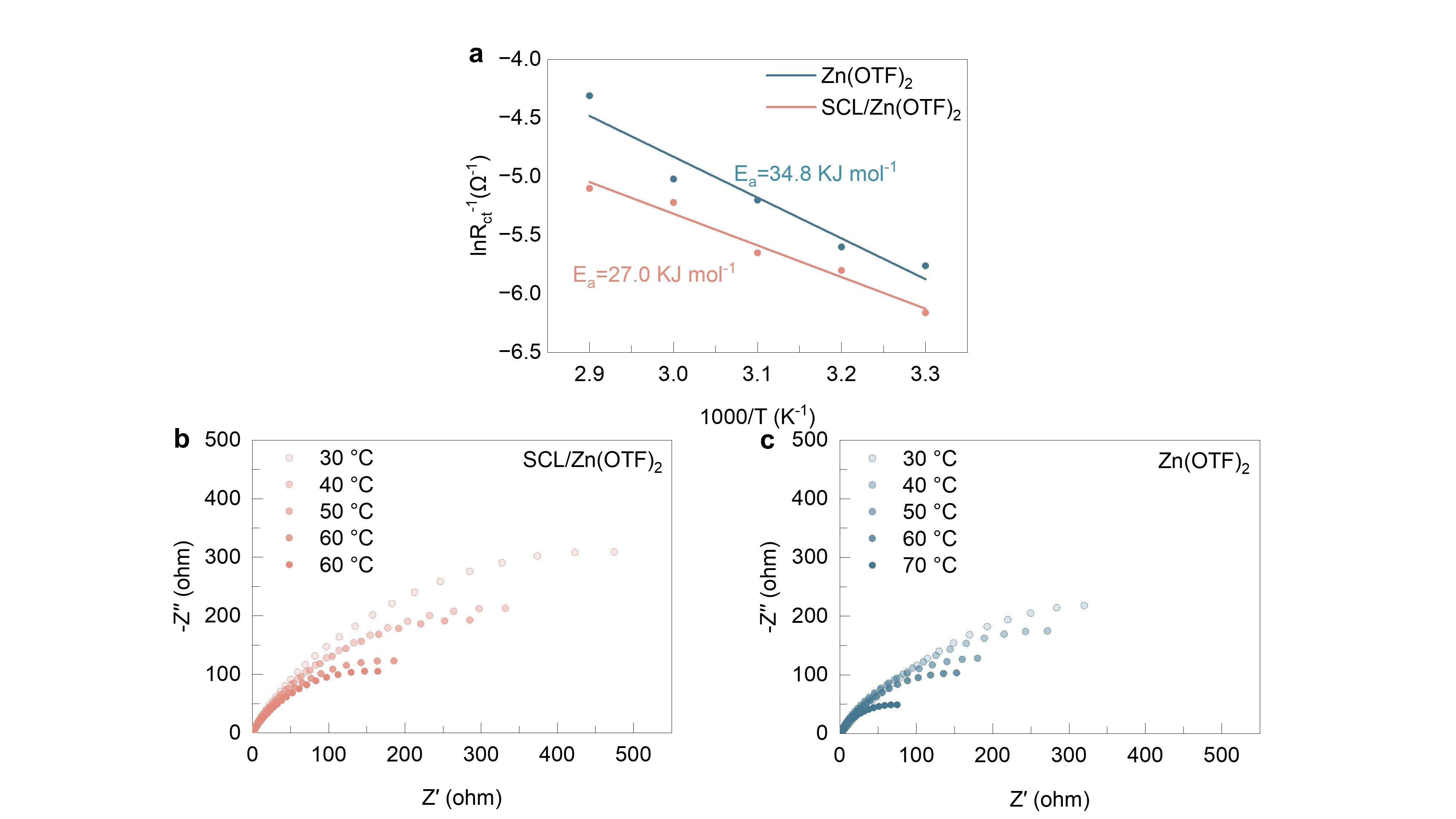


**Fig. S13 a** Arrhenius curves and activation energy (Ea) value. EIS curves under different temperatures in **a** SCL/Zn(OTF)_2_ and **b** Zn(OTF)_2_ electrolyte


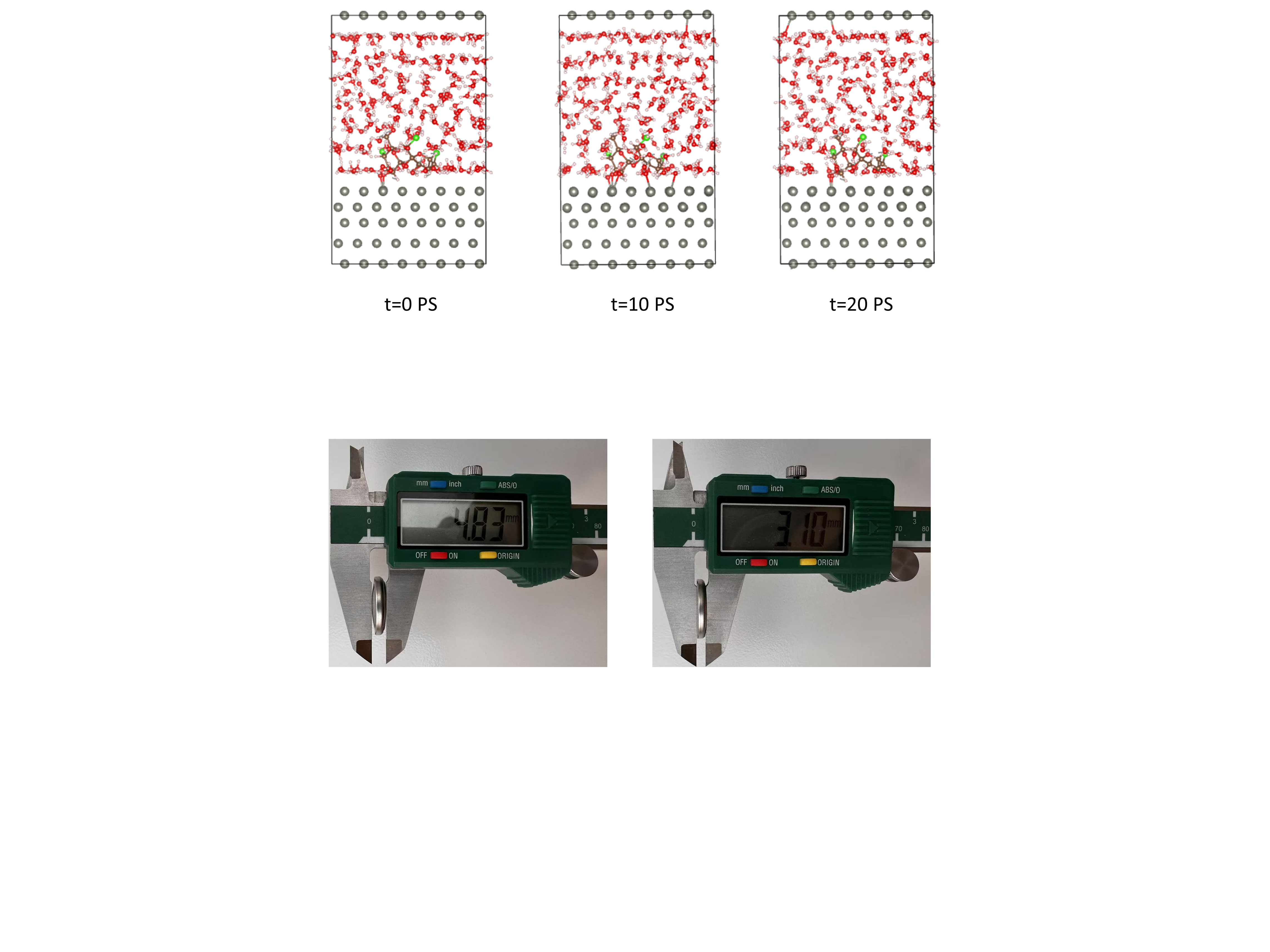


**Fig. S14** AIMD simulation for understanding the effect of water environment on SCL/Zn interaction


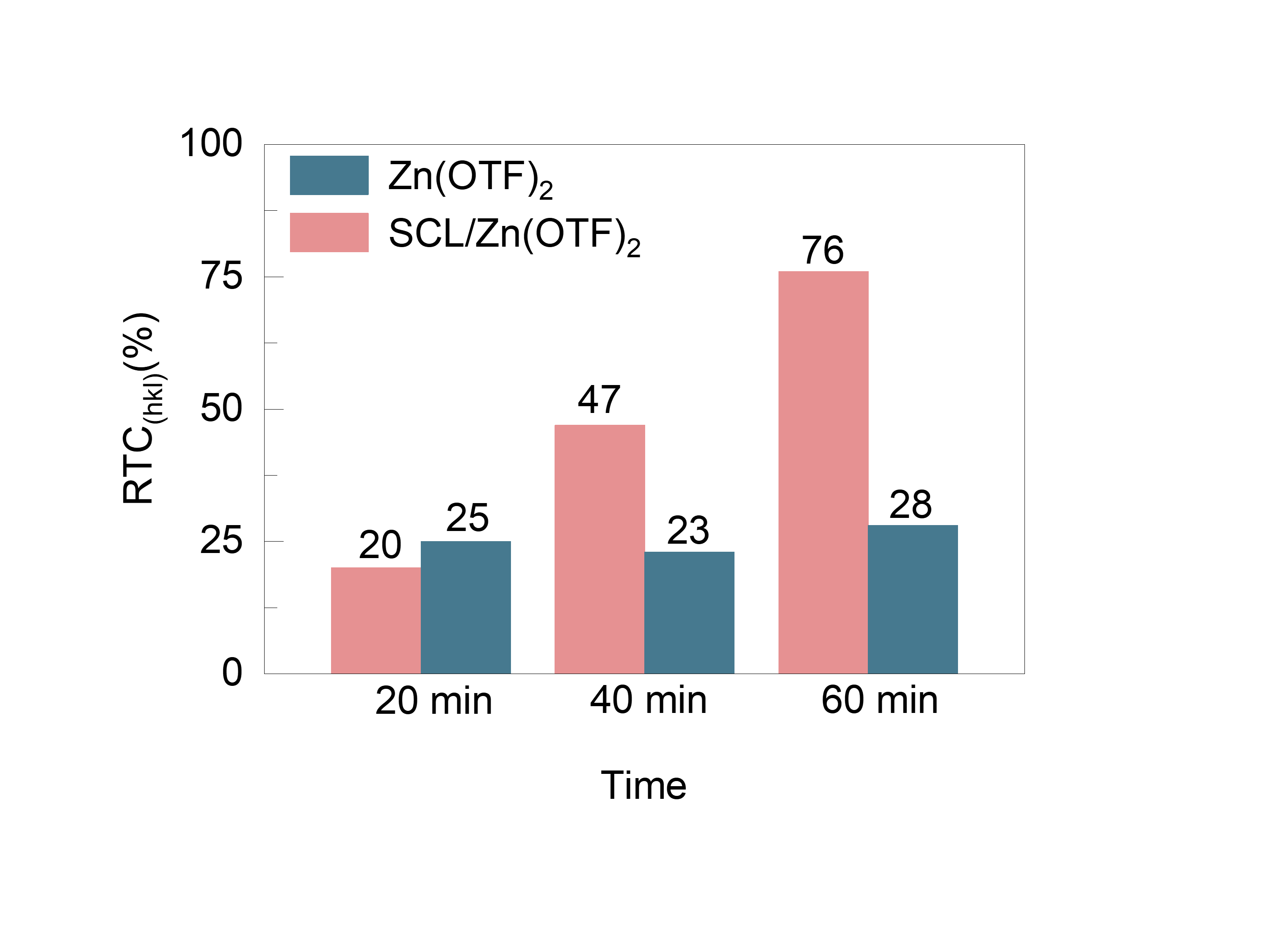


**Fig. S15** RTC value of deposited Zn anodes with different electrolytes


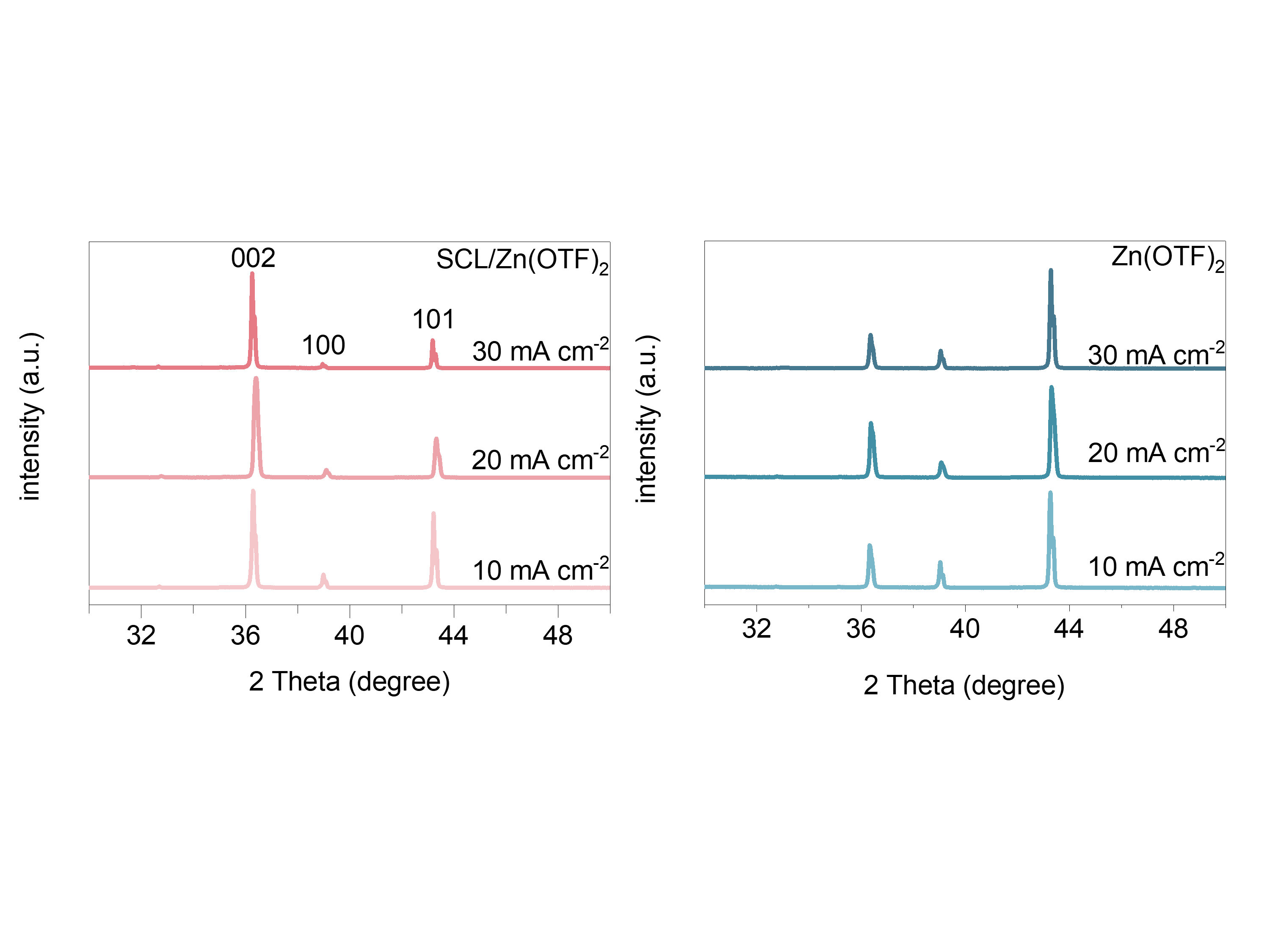


**Fig. S16** XRD spectra of the deposited Zn plates in different electrolytes at various current densities for 1h


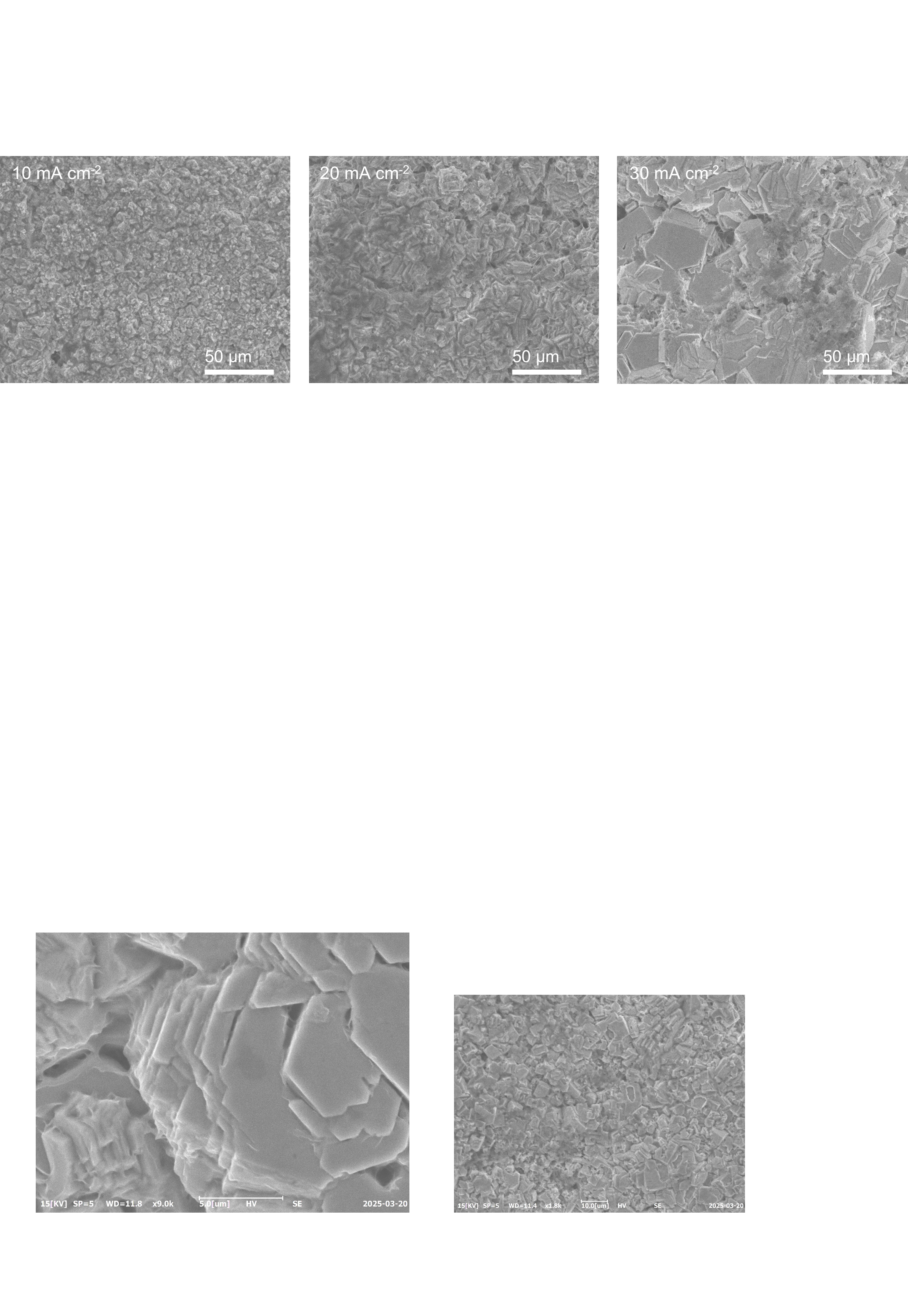


**Fig. S17** SEM images of deposited Zn anode under different current densities with fixed areal capacity of 30 mAh cm^-2^ in SCL/Zn(OTF)_2_


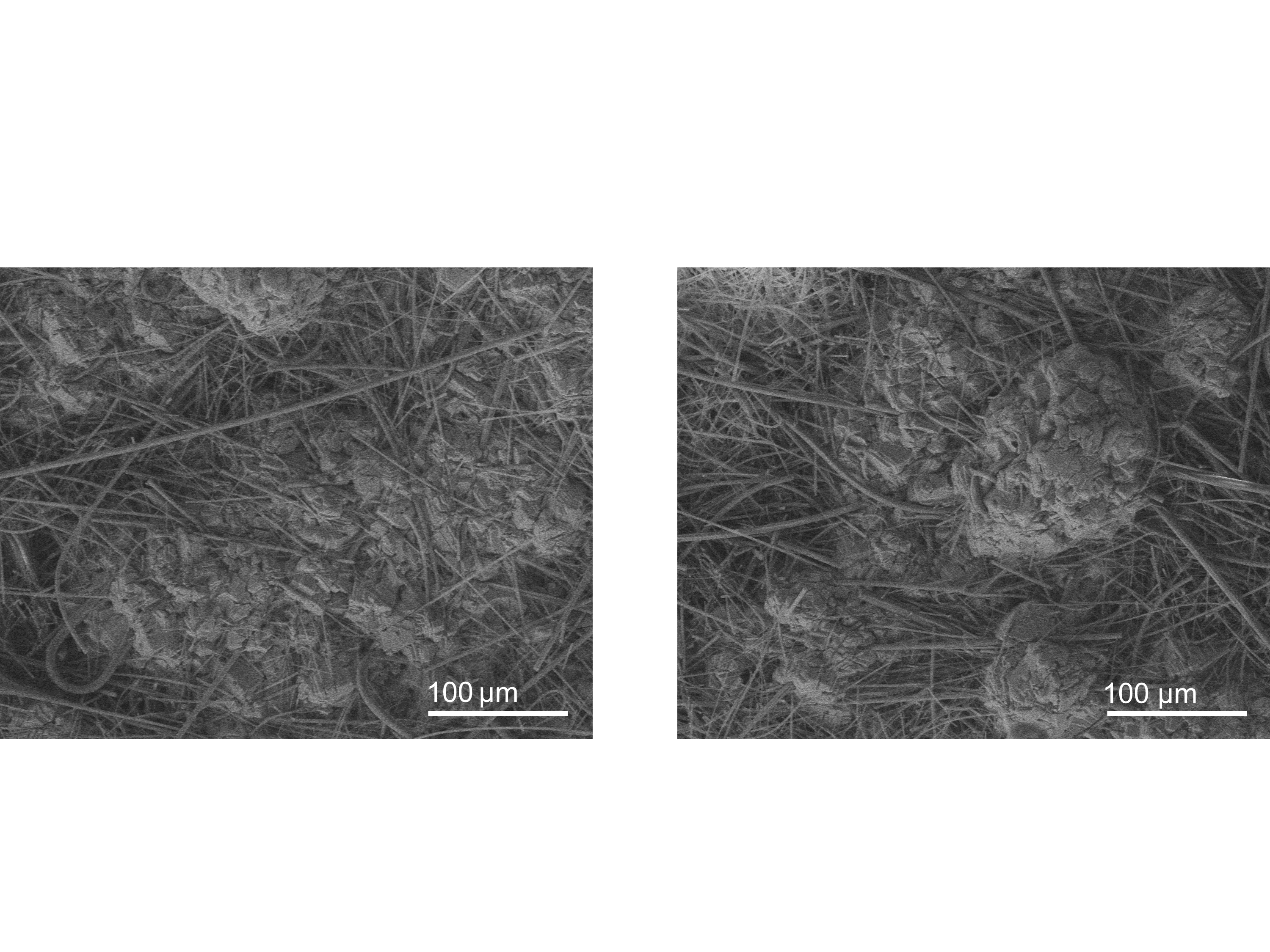


**Fig. S18** SEM image of the cycled Zn anode surface in SCL/Zn(OTF)_2_ (the left) and Zn(OTF)_2_ (the right) electrolytes after 10 cycles at 30 mA cm^-2^ and 30 mAh cm^-2^


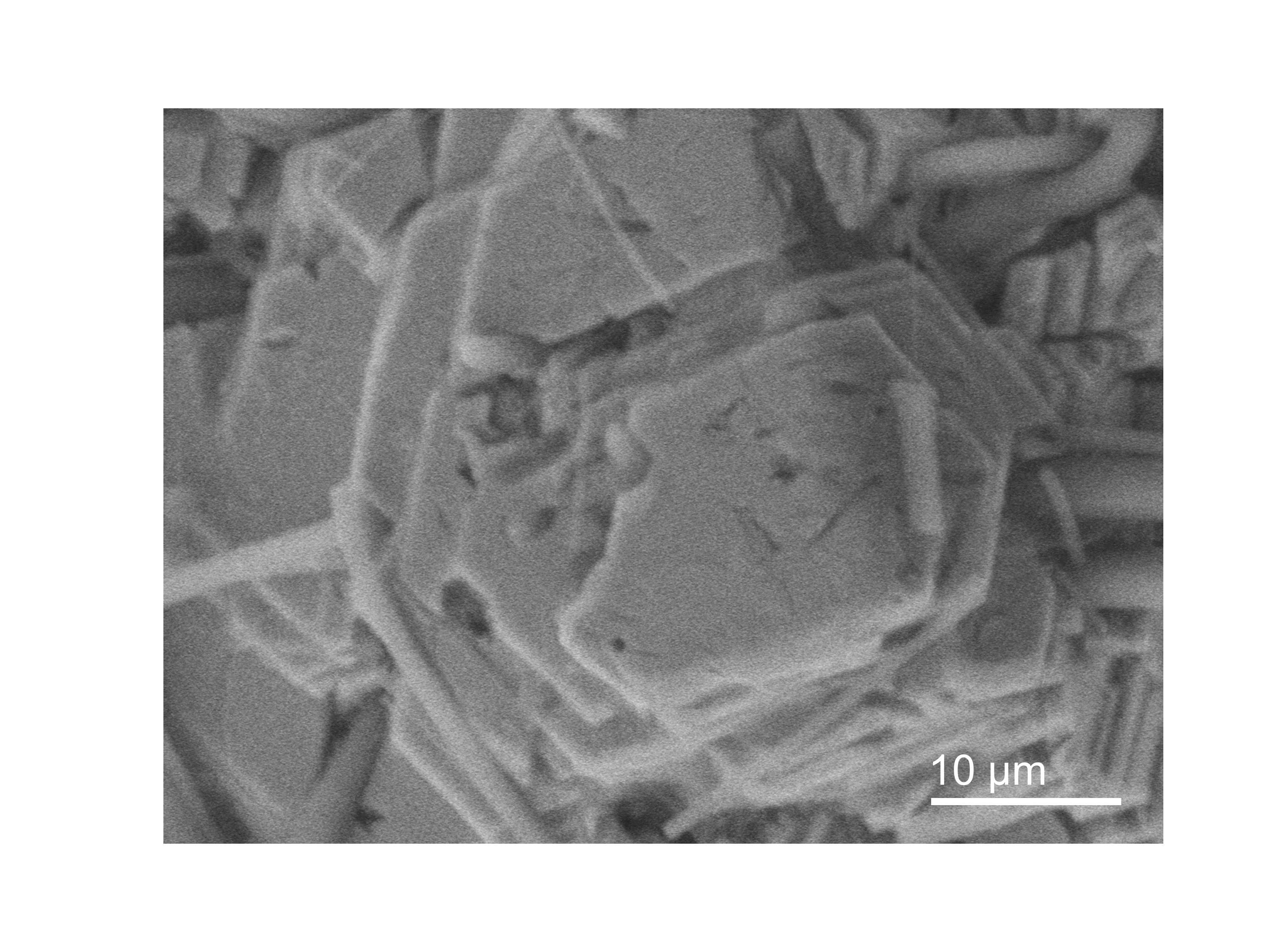


**Fig. S19** SEM image of the cycled Zn anode surface in SCL/Zn(OTF)_2_ electrolytes after 10 cycles at 30 mA cm^-2^ and 30 mAh cm^-2^


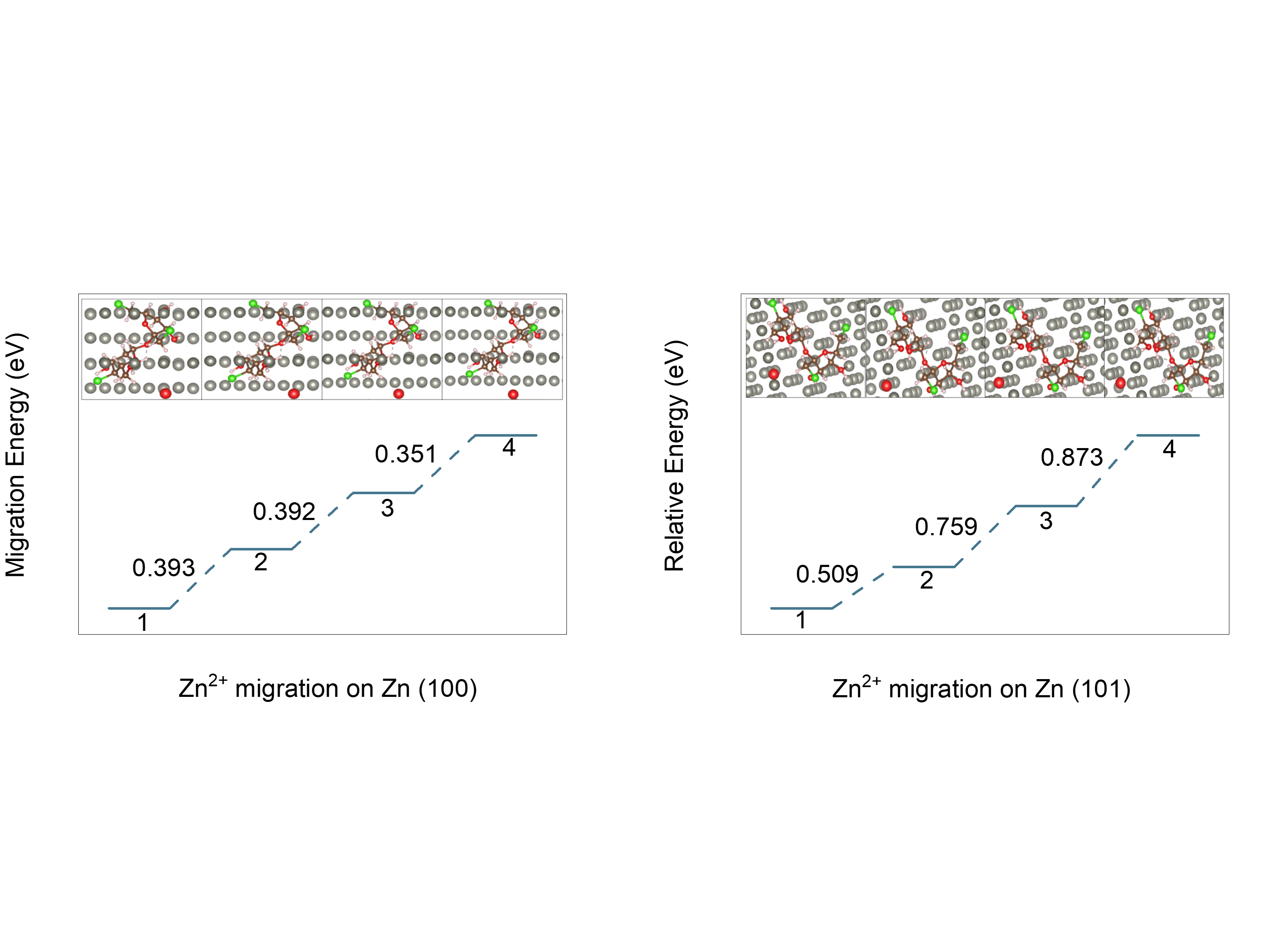


**Fig. S20** Zn^2+^ migration energy on Zn (100) and (101) after SCL adsorption, insets show the calculated migration positions


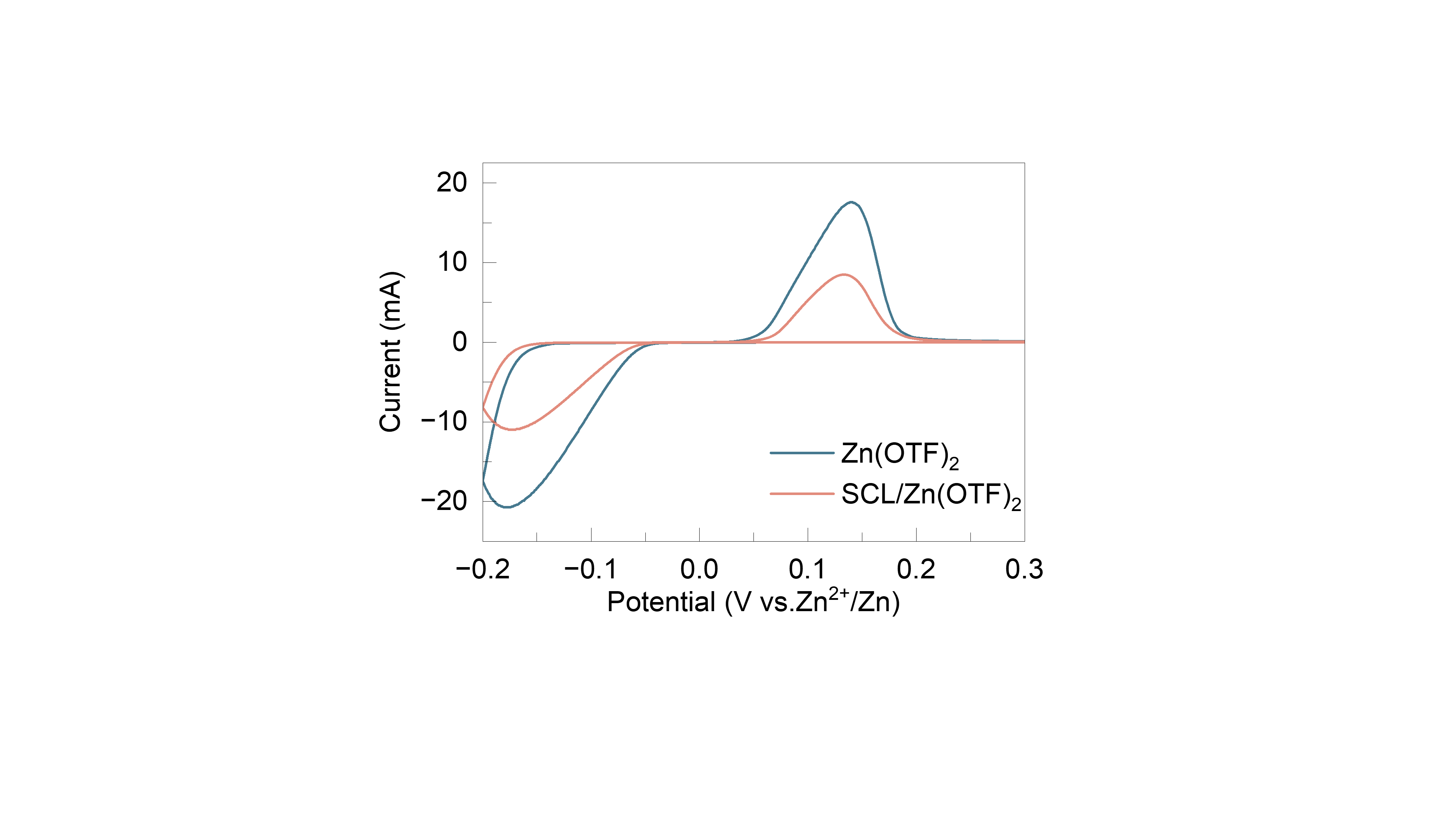


**Fig. S21** CV curves of Zn//Cu half batteries in SCL/Zn(OTF)_2_ and Zn(OTF)_2_ electrlytes


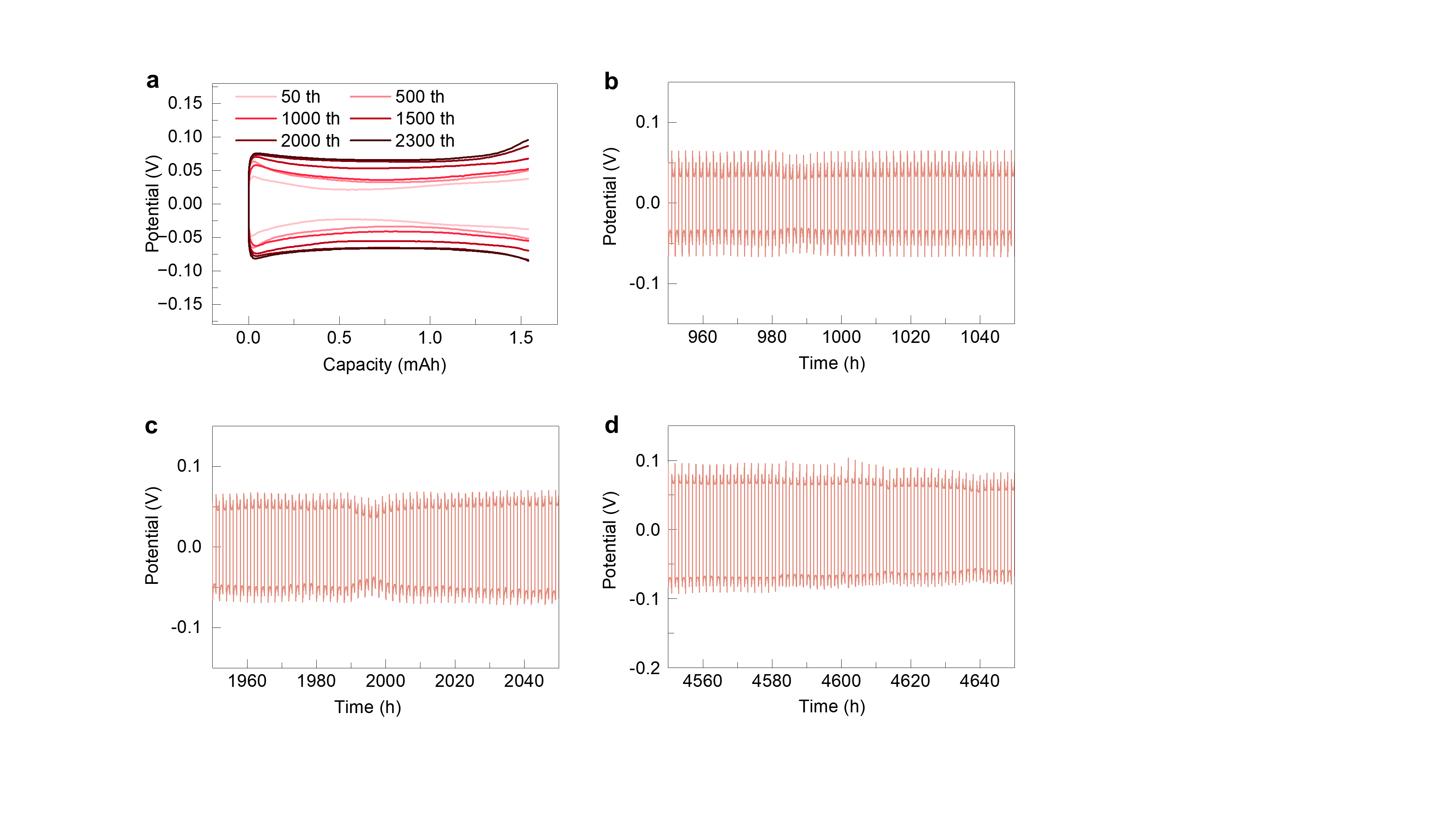


**Fig. S22 a** Potential profiles during the galvanostatic cycling at 1 mA cm^-2^, 1 mAh cm^-2^. The enlarged potential-time profiles of Zn//Zn batteries at different cycling time under the current density of 1 mA cm^-2^: **b** 950-1050 h; **c** 1950-2050 h; **d** 4550-4650 h


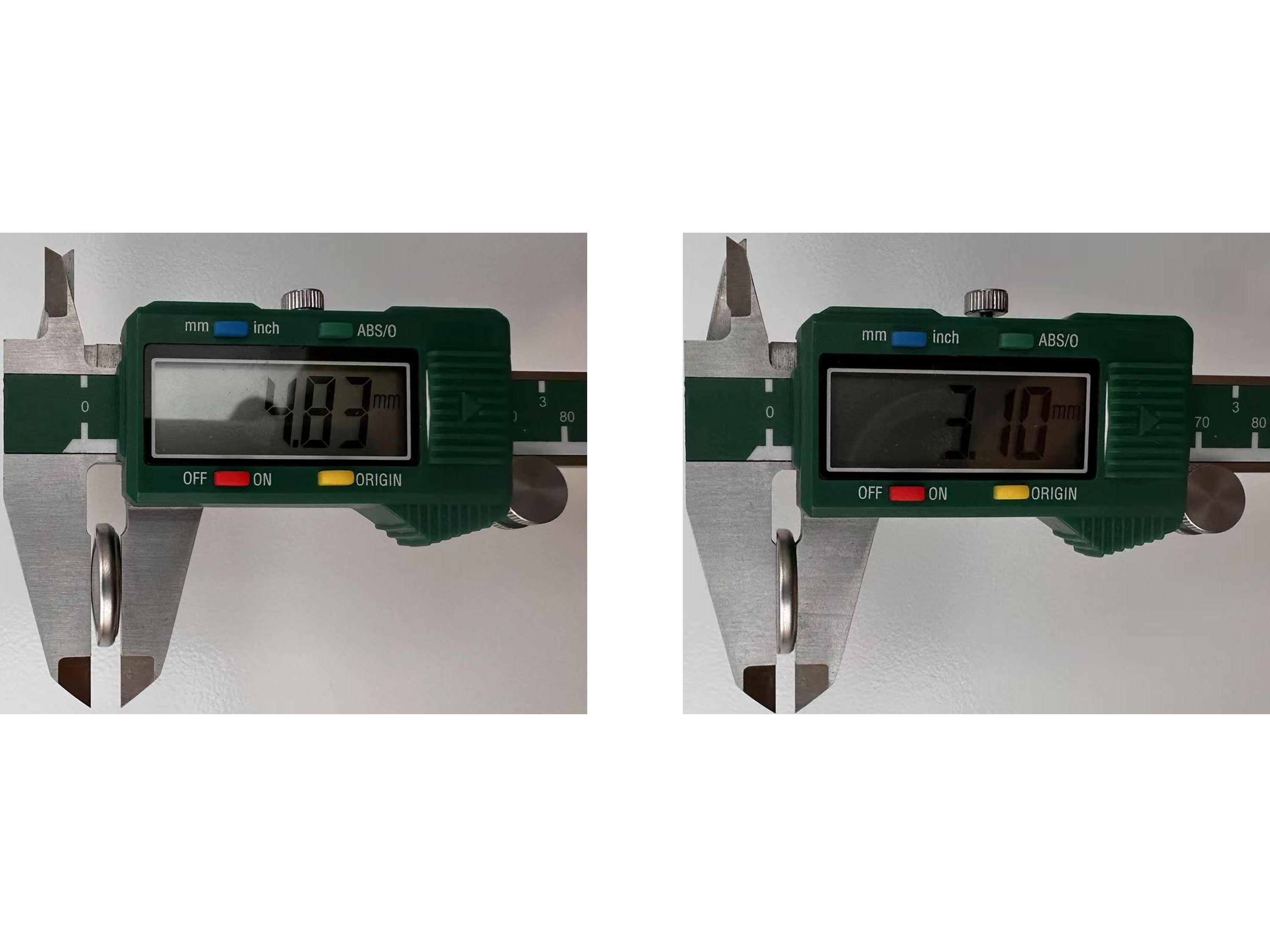


**Fig. S23** Digital photos of Zn//Zn symmetric battery operating at 30 mA cm^-2^, 30 mAh cm^-2^, after 50 cycles with SCL (the left) and after 10 cycles without SCL (the right)


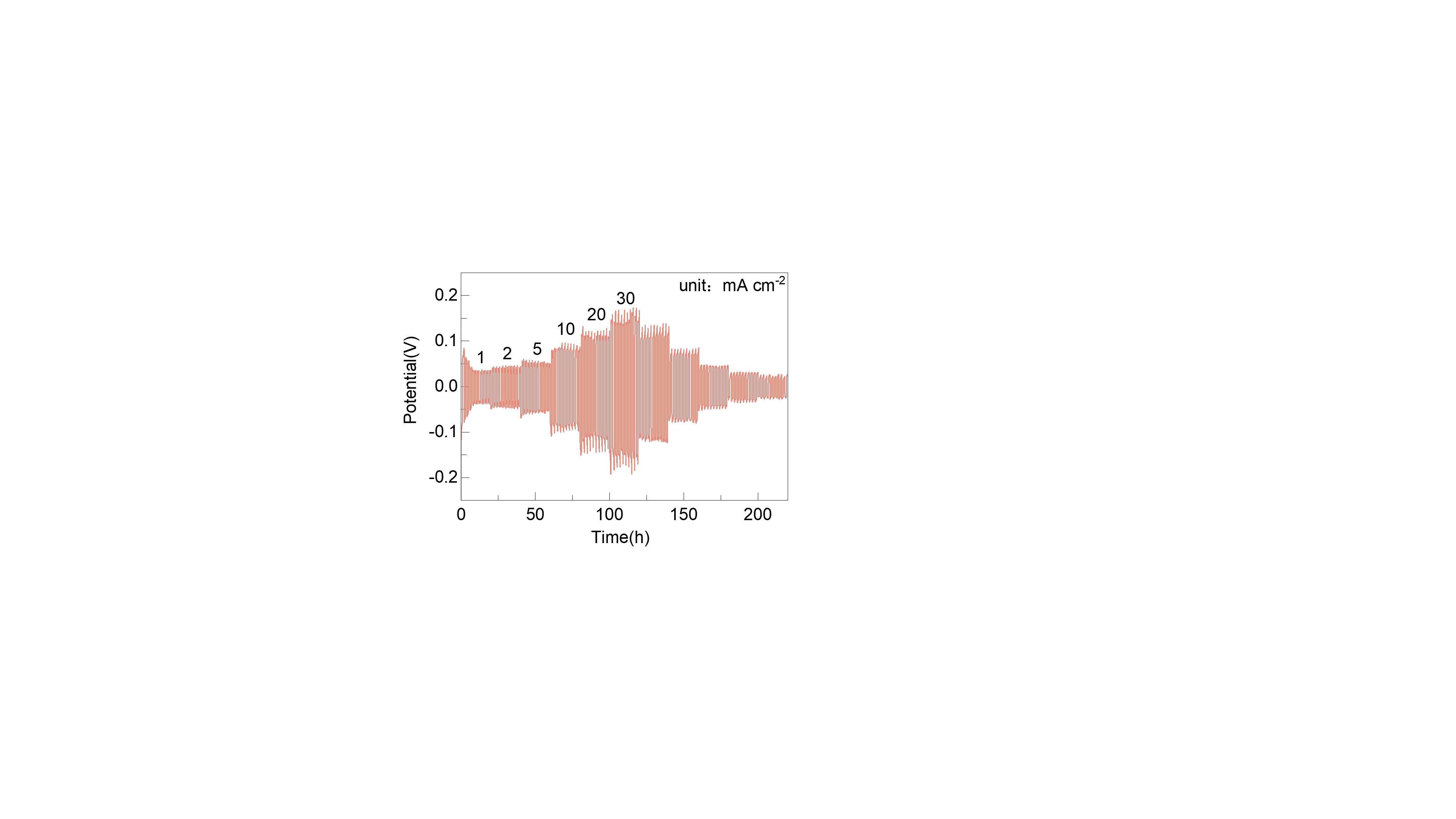


**Fig. S24** Rate cycling performance comparison of Zn//Zn symmetric batteries under different current densities and areal capacities of 1 mA cm^-2^/1 mAh cm^-2^, 2 mA cm^-2^/2 mAh cm^-2^, 5 mA cm^-2^/5 mAh cm^-2^, 10 mA cm^-2^/10 mAh cm^-2^, 20 mA cm^-2^/20 mAh cm^-2^ and 30 mA cm^-2^/30 mAh cm^-2^


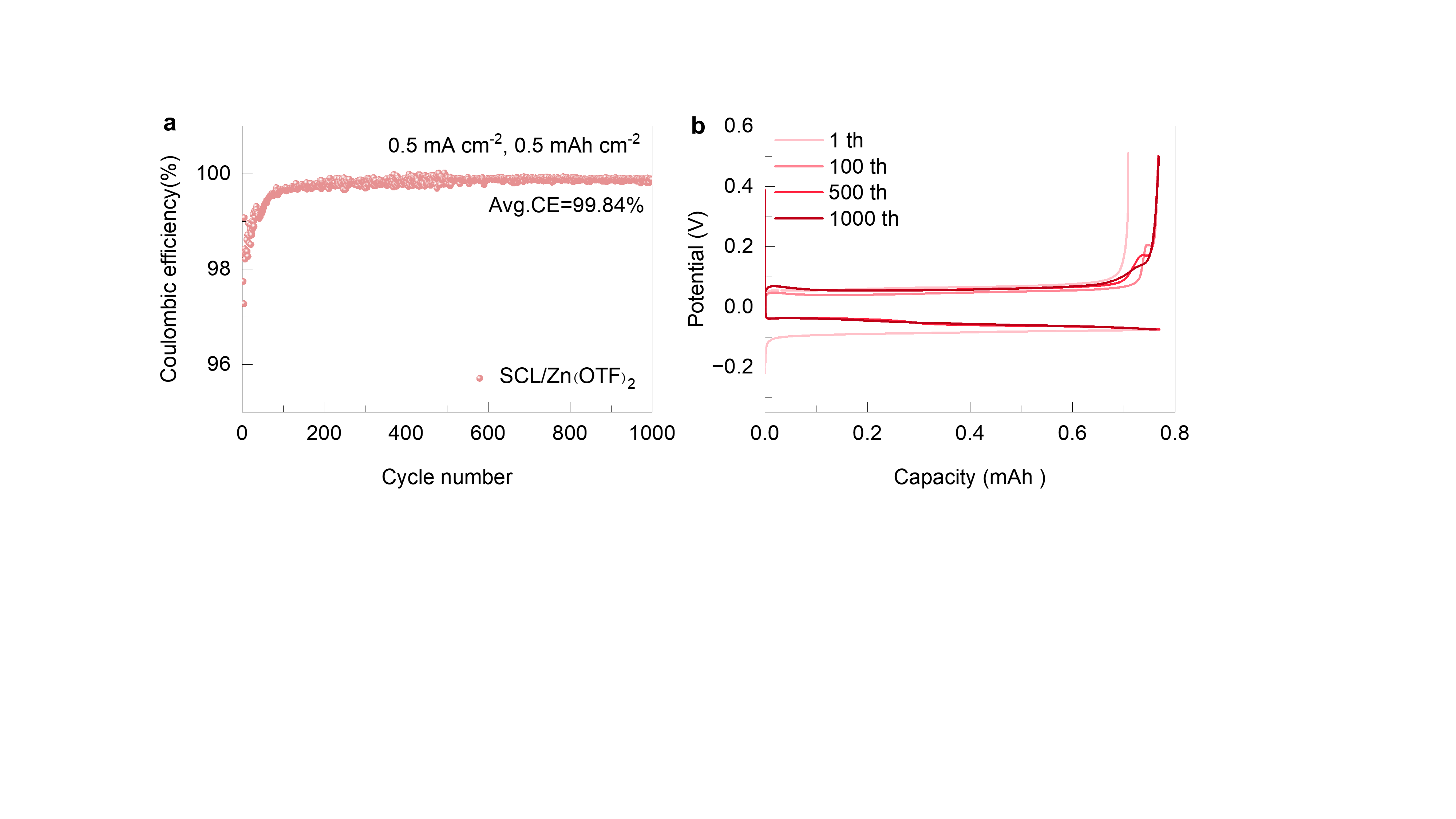


**Fig. S25** **a** CEs of Zn plating/stripping on Cu foil in SCL/ Zn(OTF)_2_ electrolyte at 0.5 mA cm^-2^ with the capacity of 0.5 mAh cm^-2^. **b** Corresponding potential-capacity profiles at different cycles


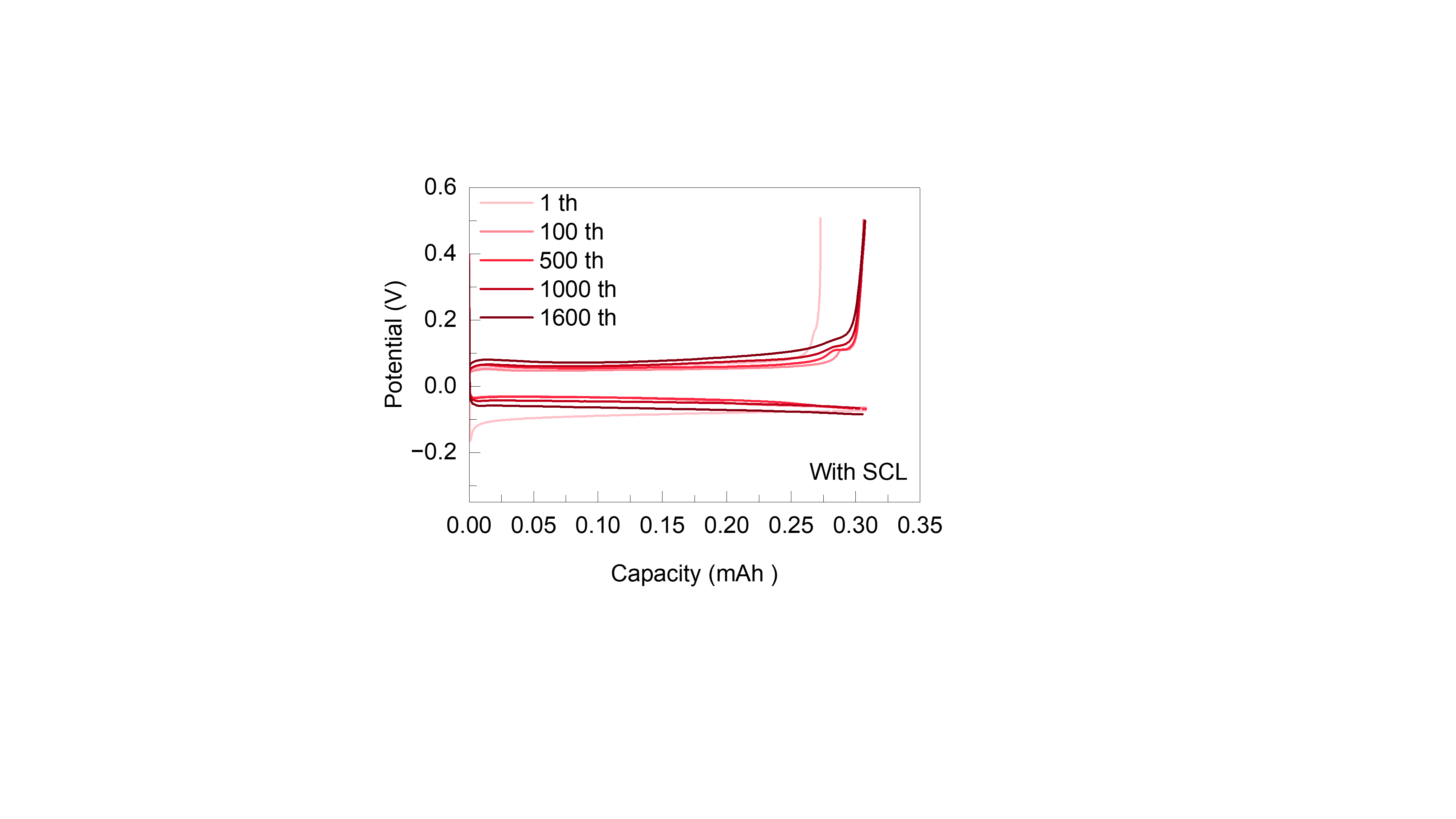


**Fig. S26** Potential-capacity profiles at different cycles at 0.2 mA cm^-2^ with the capacity of 0.2 mAh cm^-2^


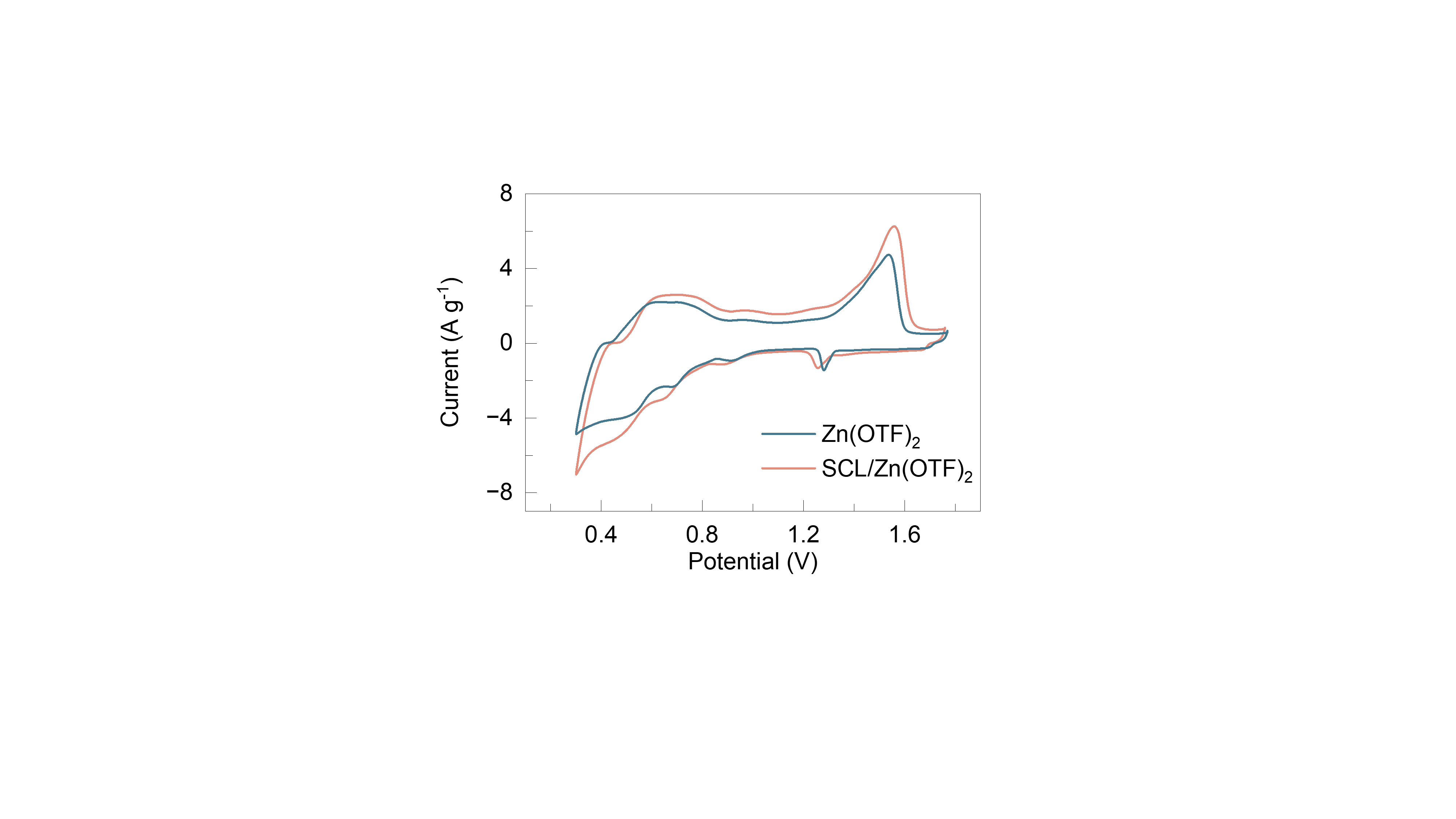


**Fig. S27** CV curves of Zn//NH_4_V_4_O_10_ full batteries with different electrolyte at 1st cycle


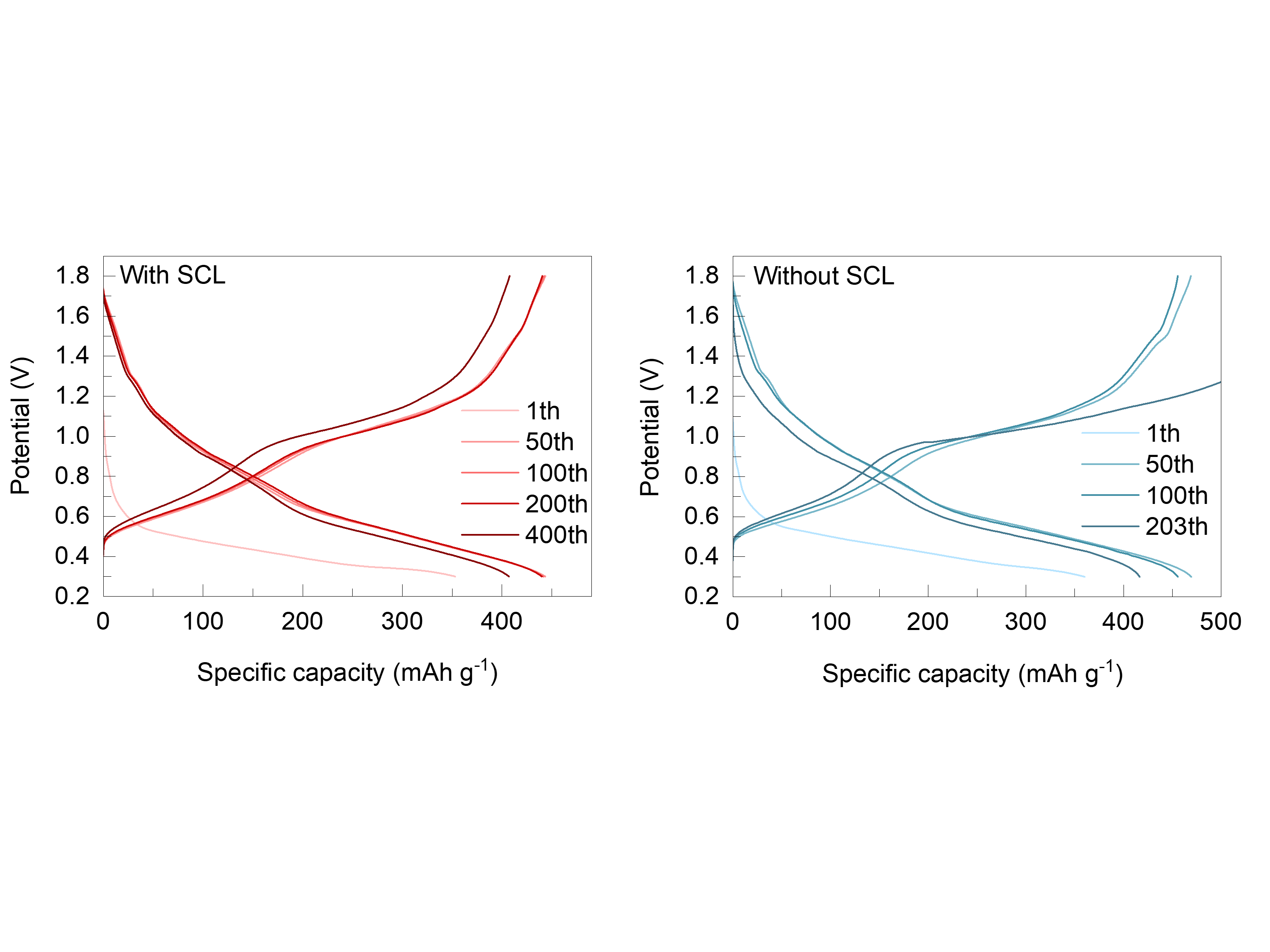


**Fig. S28** The voltage- Specific capacity profiles of Zn//NH_4_V_4_O_10_ full batteries with different electrolytes


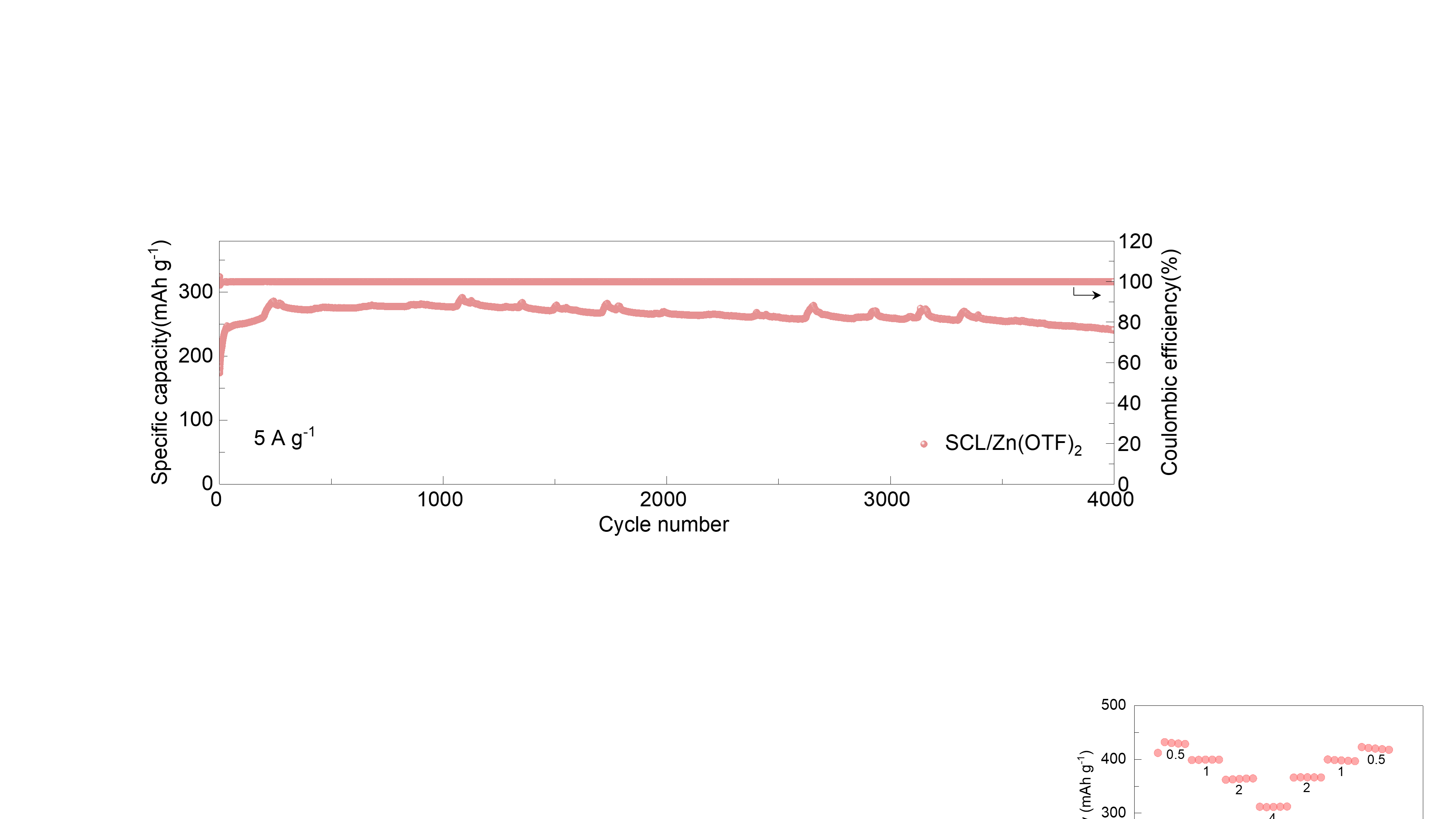


**Fig. S29** The performance of Zn//NH_4_V_4_O_10_ full battery with SCL/Zn(OTF)_2_ eletrolyte at 5 A g^-1^


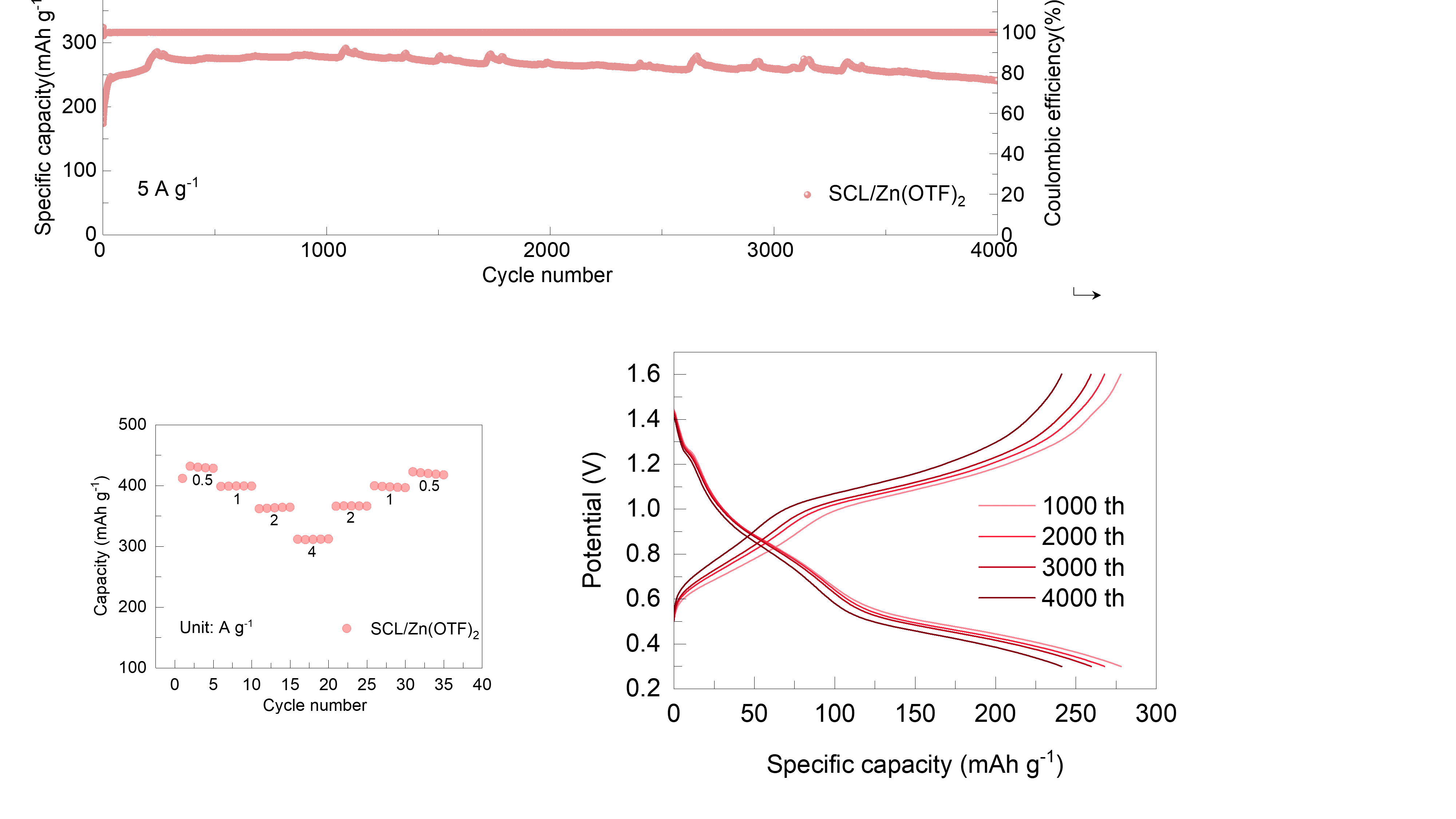


**Fig. S30** The voltage- Specific capacity profiles of Zn//NH_4_V_4_O_10_ full battery with SCL/Zn(OTF)_2_ electrolyte at 5 A g^-1^


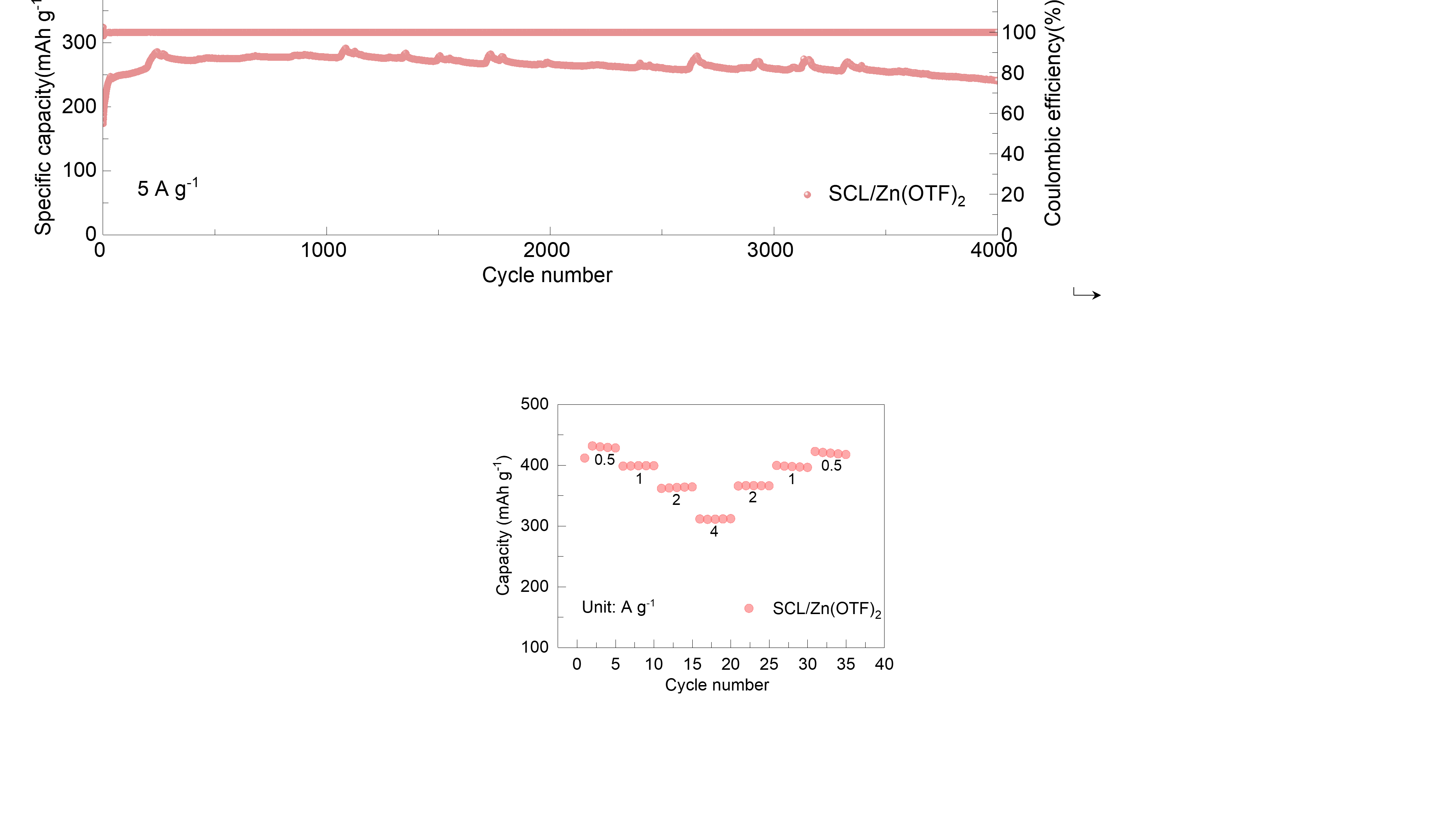


**Fig. S31** The rate performance of Zn//NH_4_V_4_O_10_ full battery with SCL/Zn(OTF)_2_ electrolyte

**Table S1** Comparison of the electrochemical stability of Zn metal electrodes

| Electrolyte | Current density areal capacity | Lifetime | Depth of discharge | References |
| --- | --- | --- | --- | --- |
| SCL/Zn(OTF)_2_ | 1 mA cm^-2^, 1 mAh cm^-2^  30 mA cm^-2^, 30 mAh cm^-2^  30 mA cm^-2^, 40 mAh cm^-2^ | 4900 h  171 h  98 h | 1.7%  73.3%  97.7% | Our work |
| LA/ ZnSO_4_ | 10 mA cm^-2^, 10 mAh cm^-2^  15 mA cm^-2^, 15 mAh cm^-2^ | 180 h  60 h | 61%  92% | [S1] |
| LA^+^/ ZnSO_4_ | 10 mA cm^-2^, 5.9 mAh cm^-2^ | 160 h | 80% | [S2] |
| DMA/ ZnSO_4_ | 1 mA cm^-2^, 1 mAh cm^-2^  30 mA cm^-2^, 30 mAh cm^-2^ | 3200 h  120 h | 3.4%  51.3% | [S3] |
| SL/ ZnSO_4_ | 24 mA cm^-2^, 24 mAh cm^-2^ | 110 h |  | [S4] |
| PMCNA/Zn(ClO_4_)_2_ | 1 mA cm^-2^, 1 mAh cm^-2^ | 4500 h |  | [S5] |
| ggg/ZnSO_4_ | 0.5 mA cm^-2^, 0.5 mAh cm^-2^ | 4500 h |  | [S6] |
| PV/ZnSO_4_ | 1 mA cm^-2^, 1 mAh cm^-2^ | 4000 h |  | [S7] |
| SAA/ ZnSO_4_ | 1 mA cm^-2^, 1 mAh cm^-2^ | 3400 h |  | [S8] |

**Table S2** The comparison of average CE with other reported works

| Electrolyte | Current density areal capacity | Coulombic efficiency | References |
| --- | --- | --- | --- |
| SCL/Zn(OTF)_2_ | 0.5 mA cm^-2^, 0.5 mAh cm^-2^  0.2 mA cm^-2^, 0.2 mAh cm^-2^ | 99.84%  99.61% | Our work |
| AS/ZnSO_4_ | 1 mA cm^-2^, 1 mAh cm^-2^ | 99.6% | [S9] |
| TG/ZnSO_4_ | 0.5 mA cm^-2^, 0.25 mAh cm^-2^ | 99.73% | [S10] |
| NCAP-Glu/ZnSO_4_ | 1 mA cm^-2^, 1 mAh cm^-2^ | 99.83% | [S11] |
| AMCA/ZnSO_4_ | 0.5 mA cm^-2^, 0.5 mAh cm^-2^ | 99.53% | [S12] |
| APM/Zn(OTF)_2_ | 0.5 mA cm^-2^, 0.5 mAh cm^-2^ | 99.16% | [S13] |
| NMS/ZnSO_4_ | 1 mA cm^-2^, 0.5 mAh cm^-2^ | 99.7% | [S14] |
| IDS/ZnSO_4_ | 1 mA cm^-2^, 1 mAh cm^-2^ | 99.5% | [S15] |

**Supplementary References**

1. M. Shi, C. Lei, H. Wang, P. Jiang, C. Xu et al., Molecule engineering of sugar derivatives as electrolyte additives for deep-reversible Zn metal anode. Angew. Chem. Int. Ed. **63**(35), e202407261 (2024). <https://doi.org/10.1002/anie.202407261>
2. R. Zhao, H. Wang, H. Du, Y. Yang, Z. Gao et al., Lanthanum nitrate as aqueous electrolyte additive for favourable zinc metal electrodeposition. Nat. Commun. **13**(1), 3252 (2022). <https://doi.org/10.1038/s41467-022-30939-8>
3. T. Wei, H. Zhang, Y. Ren, L.-E. Mo, Y. He et al., Building near-unity stacked (002) texture for high-stable zinc anode. Adv. Funct. Mater. **34**(14), 2312506 (2024). <https://doi.org/10.1002/adfm.202312506>
4. M. Wang, J. Ma, Y. Meng, J. Sun, Y. Yuan et al., High-capacity zinc anode with 96 % utilization rate enabled by solvation structure design. Angew. Chem. Int. Ed. **62**(3), e202214966 (2023). <https://doi.org/10.1002/anie.202214966>
5. D. Feng, Y. Jiao, P. Wu, Guiding Zn uniform deposition with polymer additives for long-lasting and highly utilized Zn metal anodes. Angew. Chem. Int. Ed. **62**(51), e202314456 (2023). <https://doi.org/10.1002/anie.202314456>
6. J. Zhang, Y. Liu, Y. Wang, Z. Zhu, Z. Yang, Zwitterionic organic multifunctional additive stabilizes electrodes for reversible aqueous Zn-ion batteries. Adv. Funct. Mater. **34**(34), 2401889 (2024). <https://doi.org/10.1002/adfm.202401889>
7. W. Liang, D. Li, R. Zhong, S. Tao, Y. Zhu et al., Electrolyte engineering strategy with catecholate type additive enabled ultradurable Zn anode. Adv. Funct. Mater. **35**(36), 2504195 (2025). <https://doi.org/10.1002/adfm.202504195>
8. K. Liu, M. Sun, Y. Wu, T. Zhang, A. Zhu et al., Binary electrolyte additive-reinforced interfacial molecule adsorption layer for ultra-stable zinc metal anodes. Adv. Mater. **37**(18), 2420079 (2025). <https://doi.org/10.1002/adma.202420079>
9. Z. Chen, R. Jiang, Y. Chen, H. Zhu, X. Tang et al., Zincophilic group-rich aminoglycosides for ultra-long life and high-rate zinc batteries. Energy Storage Mater. **74**, 103913 (2025). <https://doi.org/10.1016/j.ensm.2024.103913>
10. Z. Liu, R. Wang, Q. Ma, J. Wan, S. Zhang et al., A dual-functional organic electrolyte additive with regulating suitable overpotential for building highly reversible aqueous zinc ion batteries. Adv. Funct. Mater. **34**(5), 2214538 (2024). <https://doi.org/10.1002/adfm.202214538>
11. X. Fan, L. Chen, Y. Wang, X. Xu, X. Jiao et al., Selection of negative charged acidic polar additives to regulate electric double layer for stable zinc ion battery. Nanomicro Lett. **16**(1), 270 (2024). <https://doi.org/10.1007/s40820-024-01475-5>
12. N. Hu, J. Tao, Y. Tan, H. Song, D. Huang et al., Comprehensive understanding of steric-hindrance effect on the trade-off between zinc ions transfer and reduction kinetics to enable highly reversible and stable Zn anodes. Adv. Energy Mater. **14**(46), 2404018 (2024). <https://doi.org/10.1002/aenm.202404018>
13. Y. Liu, L. Miao, H. Shen, Z. Wang, K. Yao et al., Maximizing functional diversity of electrolyte additives through modular molecular engineering to stabilize zinc metal anodes. Adv. Funct. Mater. **35**(30), 2501968 (2025). <https://doi.org/10.1002/adfm.202501968>
14. Q. Zong, R. Li, J. Wang, Q. Zhang, A. Pan, Tailoring the whole deposition process from hydrated Zn^2+^ to Zn0 for stable and reversible Zn anode. Angew. Chem. Int. Ed. **63**(41), e202409957 (2024). <https://doi.org/10.1002/anie.202409957>
15. J. Yang, R. Zhao, Z. Hu, Y. Wang, K. Zhang et al., Blocking the passivation reaction *via* localized acidification and cation selective interface towards highly stable zinc anode. Energy Storage Mater. **70**, 103449 (2024). <https://doi.org/10.1016/j.ensm.2024.103449>
